# Genome-Wide Identification of the *PMEI* Gene Family in Tea Plant and Functional Analysis of *CsPMEI2* and *CsPMEI4 T*hrough Ectopic Overexpression

**DOI:** 10.3389/fpls.2021.807514

**Published:** 2022-01-27

**Authors:** Bo Li, Huan Wang, Shan He, Zhaotang Ding, Yu Wang, Nana Li, Xinyuan Hao, Lu Wang, Yajun Yang, Wenjun Qian

**Affiliations:** ^1^College of Horticulture, Qingdao Agricultural University, Qingdao, China; ^2^Engineering Laboratory of Genetic Improvement of Horticultural Crops of Shandong Province, Qingdao, China; ^3^National Center for Tea Plant Improvement, Tea Research Institute, Chinese Academy of Agricultural Sciences, Hangzhou, China; ^4^Key Laboratory of Tea Biology and Resources Utilization, Ministry of Agriculture, Hangzhou, China

**Keywords:** *Camellia sinensis*, *Arabidopsis*, pectin methylesterase inhibitor, expression patterns, biotic and abiotic stresses, overexpression, cold, flowering

## Abstract

Pectin methylesterase inhibitor (PMEI) inhibits pectin methylesterase (PME) activity at post-translation level, which plays core roles in vegetative and reproductive processes and various stress responses of plants. However, the roles of PMEIs in tea plant are still undiscovered. Herein, a total of 51 *CsPMEIs* genes were identified from tea plant genome. *CsPMEI1*-*4* transcripts were varied in different tea plant tissues and regulated by various treatments, including biotic and abiotic stresses, sugar treatments, cold acclimation and bud dormancy. Overexpression of *CsPMEI4* slightly decreased cold tolerance of transgenic *Arabidopsis* associated with lower electrolyte leakage, soluble sugars contents and transcripts of many cold-induced genes as compared to wild type plants. Under long-day and short-day conditions, *CsPMEI2*/*4* promoted early flowering phenotypes in transgenic *Arabidopsis* along with higher expression levels of many flowering-related genes. Moreover, overexpression of *CsPMEI2*/*4* decreased PME activity, but increased sugars contents (sucrose, glucose, and fructose) in transgenic *Arabidopsis* as compared with wild type plants under short-day condition. These results indicate that *CsPMEIs* are widely involved in tea plant vegetative and reproductive processes, and also in various stress responses. Moreover, *CsPMEI4* negatively regulated cold response, meanwhile, *CsPMEI2*/*4* promoted early flowering of transgenic *Arabidopsis* via the autonomous pathway. Collectively, these results open new perspectives on the roles of PMEIs in tea plant.

## Introduction

Pectin is one of three major polysaccharides in plant primary cell walls, which plays vital roles in different plant tissues and developmental stages, and also in responses to biotic and abiotic stresses. Galacturonic acid (GalA) is the main component of pectin, which can be subdivided into five classes, including apiogalacturonan (AP), homogalacturonan (HG), rhamnogalacturonan I (RG-I), rhamnogalacturonan II (RG-II), and xylogalacturonan (XGA) (Wormit and Usadel, 2018). Among them, HG is the backbone of pectin that constitutes about 65% of total pectin, and its properties could be affected by acetylating at C2–C3 atom and methylesterifing at C6 atom of GalA, respectively (Wolf et al., 2009). Within cell walls, the highly methylesterified HG could be de-methylesterified (DM) by pectin methylesterases (PMEs, E.C. 3.1.1.11) to produce negatively charged carboxyl groups and release methanol and protons (Cantarel et al., 2009). Meanwhile, PMEs activities are regulated by a type of endogenous inhibitors called pectin methylesterases inhibitors (PMEIs) (Juge, 2006).

It is now clear that PMEIs are encoded by a large multigene family both in monocotyledons and dicotyledons, and a conserved PMEI domain (PF04043) was contained in PMEI protein. In plant, the first PMEI protein (AcPMEI) was identified and purified from kiwi fruit, which has been verified to effectively repress PME activity through formation of a 1:1 non-covalent complex (Balestrieri, 1990; Di Matteo et al., 2005). Crystallographic structural analysis showed that AcPMEI is almost all helical mainly consisting of four long helices (α1–4) arranged in an anti-parallel way to form a classical up-and-down four-helical bundle (Di Matteo et al., 2005). The amino acid sequence of PMEI contains an N-terminal signal peptide and four highly conserved cysteine (Cys, C) residues. The four C residues are mainly involved in the formation of two disulfide bridges (S–S), which is critical for the stabilization of four-helical bundle structure (Wormit and Usadel, 2018). However, these structural properties are also found in a type of invertase inhibitor (INH) that shared a structural superimposition with PMEI though their sequence identity is only 20–30%. The main differences between PMEI and INH lie in the N-terminal region and the loops connecting the bundle helix. In particular, an amino acid inserted into helix α2 of INH that partially distorts the helix (Di Matteo et al., 2005). With the development of biological technology, lots of *PMEI* genes have been identified, and their functions have been explored in different plant species recently. At present, 78 *AtPMEIs* in *Arabidopsis* (Müller et al., 2013), 83 *LuPMEIs* in flax (Pinzon-Latorre and Deyholos, 2014), 49 *OsPMEIs* in rice (Nguyen et al., 2016), 95 *BoPMEIs* in *Brassica oleracea* (Liu et al., 2018a), 100 *BcPMEIs* in *Brassica campestris* (Liu et al., 2018b), 55 *SbPMEIs* in *Sorghum bicolor* (Ren et al., 2019), and 42 *PbrPMEIs* in *Pyrus bretschneideri* (Zhu et al., 2021) etc. have been identified and characterized in succession.

Until now, many research findings have revealed that PMEIs play key roles in plant vegetative and reproductive processes. With the help of mutation and overexpression techniques, the functions of many *PMEIs* have been extensively explored. In *Arabidopsis*, there have been more than 12 *AtPMEIs* verified to mediate plant growth and development. Among them, AtPMEI1 (At1g48020) and AtPMEI2 (At3g17220) inhibited plant-derived PME activity rather than microbial PME activity, which shared 38% sequence identity with AcPMEI from kiwi fruit (Raiola et al., 2004). Similarly, a functional PMEI of *Vitis vinifera*, VvPMEI1, also typically inhibited the plant PME activity but not microbial PME activity (Lionetti et al., 2015). In addition, the recombinant OsPMEI28 protein showed high inhibitory activity against PME protein, overexpression of *OsPMEI28* caused dwarf phenotypes and reduced culm diameter in transgenic rice lines (Nguyen et al., 2017). Similarly, overexpression of *AtPMEI3* resulted in hyper-methyl-esterification of HG and effected the formation of flower primordia (Lionetti et al., 2007). The expression of *AtPMEI6* showed a spatio-temporal pattern in seed coat epidermal cells, and overexpression of *AtPMEI6* inhibited the endogenous PME activity and decreased the total methylesterification of mucilage fractions and demucilaged seeds in transgenic plants (Saez-Aguayo et al., 2013). GUS staining results showed that the promoter of a flower-specific gene, *SlPMEI*, expressed specifically in mature pollen of tomato and reproductive organs of transgenic *Arabidopsis* (Raiola et al., 2004).

In addition to mediate plant growth and development, PMEIs have also been reported to participate in defending pathogen infection and responding to environment stresses. In *Arabidopsis*, the expressions of three PMEIs genes, *AtPMEI10*/*11*/*12*, were induced by *Botrytis cinerea* infection mainly through jasmonic acid and ethylene signaling. Moreover, the reverse genetic approach results found that the PME activity was increased but the DM of pectin was decreased in *pmei10*/*11*/*12* mutants, respectively, which finally increased lesion formation during *B. cinerea* infection. These results indicated that AtPMEI10/11/12 served as mediators to maintain cell wall integrity in plant immunity (Lionetti et al., 2017).

Tea plant (*Camellia sinensis*) is a type of evergreen economic plant, which is appropriate to growth at normal temperature, high humidity and acid soil (pH 5.5) environments. However, with the frequency of extreme climate in recent years, tea plants usually cannot overwinter safely or grow normally when suffered from drought, chilling, freezing, or cold spell damages. Therefore, more and more studies have been focused on the molecular mechanisms of tea plant stress responses. At present, many functional genes involved in vegetative (Xia et al., 2021), reproductive (Jing et al., 2020), nutrient uptake (Arkorful et al., 2020), secondary metabolism (Yu et al., 2021; Zhao et al., 2021), and stress response (Qian et al., 2018; Wang et al., 2018; Yao et al., 2020) have been extensively explored. However, the functions of PMEIs in tea plant still remain unknown. Herein, we performed a genome-wide identification and characterization analysis of *CsPMEIs*, analyzed the tissue-specific profiles and spatio-temporal patterns of four *CsPMEIs* (*CsPMEI1*-*4*), and finally explored the functions of *CsPMEI2*/*4* in transgenic *Arabidopsis*, respectively. Our study opened the door for the functional study of pectin in tea plant, and these results also provided a firm foundation for deeply exploring the functions of PMEs and PMEIs in tea plant.

## Materials and Methods

### Plant Materials and Multiple Treatments

For tissue-specific analysis, the apical buds, the first leaves, the second leaves, the third leaves, young fruits, mature fruits, young stems, flowers, and roots were sampled from 4-year-old clonal potted seedlings of the ‘LongJing43’ cultivar during flowering season (October). Each tissue contains three independent biological replicates, and all tissues were frozen in liquid nitrogen and then stored at −80°C until used.

For various abiotic stress treatments, 1-year-old clonal hydroponic seedlings of the ‘LongJing43’ cultivar with the similar growth potential were used for 4°C, PEG-6000 [10% (w/v)], 150 mmol⋅L^–1^ NaCl and 100 μmol⋅L^–1^ ABA treatments. The detailed treatment methods were carried out as described by Qian et al. (2016). The nutrient solution formulation was shown in Supplementary Table 1. Five-year-old clonal seedlings of the ‘LongJing43’ cultivar at one bud and five leaf stages were used for *Colletotrichum fructicola* (*C. fructicola*) infection. The detailed infection method was performed as described by Wang et al. (2016). 15-year-old of four-tea cultivars, including two cold-resistant cultivars (‘ZheNong113’ and ‘LongJing43’) and two cold-susceptible cultivars (‘ZheNong12’ and ‘DaMianBai’) as demonstrated by Wang et al. (2019), were used for cold acclimation (CA) analysis in 2018–2019. The sampling methods were performed according to the methods stated by Qian et al. (2018). 15-year-old clonal tea plants of the ‘LongJing43’ cultivar that planted in field were used for bud dormancy analysis. The sampling methods were implemented as described by Hao et al. (2018). The above mentioned treatments were, respectively, performed three independent biological replicates, and all samples were quickly frozen in liquid nitrogen and then stored at −80°C until used.

2-year-old clonal potted cuttings of the ‘LongJing43’ cultivar at two different growth stages, including one bud and one leaf (OBOL) stage and one bud and three leaves (OBTL) stage, were used for cold treatment. Before proceeding, the cuttings were moved into the greenhouse for normal culture. The growth conditions were as follows: lighting time, 14 h/10 h (light/dark); temperature, 23°C; and humidity, 75%. When the cuttings reached the standards, the temperature of greenhouse was plummeted to 4°C without changing lighting time and humidity. The cuttings with OBOL were continued for 3 days at 4°C, and the samples (OBOL) were collected at 0, 1, 3, 6, 12, 24, 48, and 72 h, respectively. Similarly, the cuttings with OBTL were continued for 8 days at 4°C, and the samples (OBTL) were collected at 0, 1, 2, 3, 4, 5, 6, 7, and 8 days, respectively. Subsequently, the cuttings were returned to 25°C for 2 days, and the samples (OBTL) were collected at 1 and 2 days. All samples were immediately frozen in liquid nitrogen and then stored at −80°C until used. Each sampling time point was performed five biological replicates, and each biological replicate contains 10 pots of tea plants and each pot contained three cuttings.

One-year-old clonal hydroponic cuttings of the ‘LongJing43’ cultivar were used for sugar treatment. 3% sucrose (Suc), 3% glucose (Glu), 3% fructose (Fru), and 3% mannose (Man) treatments were, respectively, performed as described by Qian et al. (2018). Each sampling time point was performed three independent biological replicates, and each biological replicate contains four pots of tea plants and each pot contains eight cuttings. The nutrient solution formulation was shown in Supplementary Table 1.

The wild-type (WT) *Arabidopsis thaliana* (*Columbia*-0 ecotype) was used to construct overexpression (OE) lines of the opening reading frame of *CsPMEI2* and *CsPMEI4*. The seeds of the homozygous OE lines and WT were surface sterilized and vernalized for 2 days, and then sown on 1/2 MS medium (1.5% Suc, 0.8% Agar, pH 5.7) for 2 weeks. Thereafter, the seedlings were transplanted on the seedling block in the growth chamber under short-day (SD) (8 h light at 22°C/16 h dark at 20°C) or long-day (LD) (16 h light at 22°C/8 h dark at 20°C) conditions. The light intensity was 100 μmol/m^2^s both under SD and LD conditions. The OE lines of blank vector were omitted in this study as a same phenotype detected in WT as demonstrated by Qian et al. (2018). Under LD condition, 4-week-old seedlings of both WT and *CsPMEI2*/*4*-OE lines were sampled for analyzing the expressions of flowering-related genes. Under SD condition, 10-week-old seedlings of both WT and *CsPMEI2*/*4*-OE lines were sampled for measuring PME activities, sugars (sucrose, glucose, and fructose) contents, and flowering-related genes expressions.

### Genome-Wide Identification of *CsPMEIs*

The hidden Markov models (HMM) file of PMEI domain (PF04043) downloaded from Pfam database^1^ was used to obtain CsPMEIs similar sequences from the tea plant protein database of ‘ShuChaZao’ cultivar (Wei et al., 2018) by using HMMER 3.0 software with the default parameters. Subsequently, all of these sequences were performed alignment analysis to delete the sequence that contains only one or two conserved Cys residues. The redundant sequences were submitted to the SMART server^2^ and the Conserved Domain Database of NCBI^3^ for verifying whether they had conserved the PMEI domain. Finally, the sequences that contain only one conserved PMEI domain were retained for further analyses.

### Bioinformatics Analysis of *CsPEMIs* in Tea Plant

The bioinformatics of *CsPEMIs*, including ORF lengths, amino acid numbers, molecular weights, signal peptides, transmembrane regions, and subcellular locations were performed as described by Wang et al. (2021). For phylogenetic analysis, 262 PMEI domain contained proteins (Supplementary Table 2), including 78 AtPEMIs from *Arabidopsis*, 55 SbPEMIs from *Sorghum bicolor*, 55 PbrPEMIs from pear, 2 AcPEMIs from kiwi fruit, 51 CsPEMIs from tea plant and 21 INHs, were used to generate phylogenetic tree by MEGA 7.0 software. The parameters were set as follows: maximum likelihood method, 1,000 bootstrap replications, Jones–Taylor–Thornton (JTT) model, and partial deletion. Thereafter, the generated tree was uploaded to the ITOL website^4^ for further beautification. The exon–intron structures of *CsPMEIs* were predicted by using GSDS 2.0 website^5^. The *cis*-acting elements of 2,000-bp promoter sequences of *CsPMEIs* were predicted by using PlantCARE website^6^. Multiple amino acids sequences alignment analysis was carried out by using Clustlx2.0 software, and then output the result with the help of GENEDOC software.

### Cloning the Full-Length of *CsPMEIs*

Before the tea plant genome sequenced, 20 expressed sequence tags (EST) that annotated as PMEI domain containing proteins were identified from the transcriptome data of the tea plant under CA condition (Wang et al., 2013). After assembled by Seqman software, a total of 4 contigs were obtained and served as templates to design RT-PCR primers for TA cloning. The TA cloning method was performed as described by Qian et al. (2016). The amplified and purified PCR products were inserted into the *pEASY*-*Blunt Zero* vector (TransGen Biotech, Beijing, China), then transferred into *Trans5α* chemically competent cell (TransGen Biotech, Beijing, China) and sequenced finally. All RT-PCR primers were listed in Supplementary Table 3.

### Vector Construction, Plant Transformation, and Cold Treatment of Transgenic Plants

The vector construction and plant transformation methods were carried out as stated by Qian et al. (2018) with some modification. The Gateway technology was used to construct overexpression vectors (Landy, 1989). Simply, the ORF of *CsPMEI2* and *CsPMEI4* without stop codons were firstly cloned into the entry vector (*pENTR/D-TOPO*) (Invitrogen, CA, United States) following the instruction of manufacturer, respectively. After verified by sequencing, the ORF of *CsPMEI2* and *CsPMEI4* were transferred into the destination vector (*pH7FWG2*) by using LR Clonase II enzyme mix kit (Invitrogen, Carlsbad, CA, United States) (Ishimaru et al., 2005), respectively. The plasmids of recombined vectors of *CsPMEI2* and *CsPMEI4* were separately mobilized into *Agrobacterium tumefaciens* strain *GV3101*, and finally transformed into *Arabidopsis* via *Agrobacterium*-mediated floral infiltration (Clough and Bent, 2010). The positive *CsPMEI2*-OE lines and *CsPMEI4*-OE lines were obtained by *hygromycin* B screening, and the transcript abundances of *CsPMEI2* and *CsPMEI4* in each OE-line were quantified by qRT-PCR. Finally, three OE-lines with different transcript abundances of *CsPMEI2* and *CsPMEI4* were, respectively, used to proceed further experiments. The primers used for vector construction were listed in Supplementary Table 3.

### Cold and ABA Treatments of Transgenic Plants

For cold treatment, the seeds of WT plants and three *CsPMEI4*-OE lines (OE4-2, OE4-7, and OE4-11) were sterilized and vernalized firstly, and then they were sown onto 1/2 MS medium for germinating in the growth chamber with photoperiod (10 h light at 22°C/14 h dark at 20°C) and 100 μmol/m^2^s. Two weeks later, the seedlings with four rosette leaves were transplanted on the seedling blocks and grown in the growth chamber for another 2 weeks. For cold treatment, both WT plants and *CsPMEI4*-OE plants were treated at 4°C for 7 days without changing the light time and intensity. The rosette leaves were collected to measure total soluble sugar (TSS) and the expressions of cold-responsive genes. To detect electrolyte leakage, 4-week-old seedlings of both WT plants and *CsPMEI4*-OE plants were exposed to −6°C for 8 h without changing the light time and intensity. The control seedlings were grown normally in the growth chamber. Each treatment contained three independent biological replicates, and each replicate contained six seedlings of each OE lines and WT plants, respectively.

ABA treatment was performed as described by Jing et al. (2020). Two-week-old seedlings of *CsPMEI2/4*-OE lines and WT plants were sprayed with a 20 μM ABA solution twice a week until flowering under LD condition. Meanwhile, the controls were sprayed with distilled water. The phenotypes, flowering time, and leaf number were recorded. Each treatment contained three independent biological replicates, and each replicate contained fifteen seedlings of each OE lines and WT plants, respectively.

### Flowering Time, Leaf Number, and Plant Height Measurements

The methods used for evaluating the flowering time and other phenotypes were conducted as described by Jing et al. (2020). The WT and OE-lines were cultured in seedling blocks under LD and SD conditions, respectively. The time from seed sterilization to floral bud formation was recorded as flowering time, and the total number of rosette leaves were counted at the same time. The plant heights of WT and OE-lines were measured once the plants stop flowering. The sizes of the leaves, flowers and seeds of WT and OE-lines were also surveyed as described by Wang et al. (2012). Three independent biological replicates were performed, and each replicate contained fifteen seedlings of each OE lines and WT plants, respectively.

### Electrolyte Leakage and Sugar Contents Measurements

The electrolyte leakage (EL) was assayed as described by Qian et al. (2018). For detecting soluble sugars contents, 0.1 g fresh sample was extracted with 1.0 mL extraction buffer in a pre-cooled mortar on ice box, and then transferred into 1.5 mL microcentrifuge tube to water bath at 80°C for 10 min. After centrifuged at 25°C, 4,000 × *g* for 10 min, the supernatants were decolorized. Thereafter, 1.0 mL extraction buffer was added and centrifuged at 25°C, 4,000 × *g* for 10 min again. The supernatant was used for total soluble sugar (TSS), Suc, Glc, and Fru measurements according to the instructions of the corresponding sugar measurement kits (Suzhou Comin Biotechnology, Suzhou, China), respectively.

### Pectin Methylesterase Activity Measurements

Pectin methylesterase activity was assayed by using NaOH indirect titration method as the manufacture’s introduction (Suzhou Comin Biotechnology, Suzhou, China). 1 g sample was thoroughly ground in an ice bath with 2 mL pre-cooled extraction buffer. After centrifuged at 4°C, 12,000 × *g* for 10 min, the supernatant was used.

### Quantitative Real-Time RT-PCR Analysis

Total RNA isolation, first-strand cDNA synthesis and qRT-PCR analysis of all samples were conducted as demonstrated by Qian et al. (2018). The qRT-PCR reaction system was mixed as follows: 5.0 μL SYBR Premix Ex *Taq*, 1 μL cDNA, 0.8 μL forward/reverse primers, and 3.2 μL distilled water. The qRT-PCR program was run as follows: 95°C, 15 s for predegeneration; then 94°C, 5 s and 58°C, 30 s for amplification with 40 cycles; finally, a melting curve was added. Two housekeeping genes, *CsPTB* of tea plant (Hao et al., 2014) and *AtEF* of *Arabidopsis* (Yuan et al., 2008), were, respectively, used to quantify the relative expression levels of the target genes based on 2^–Δ^ *^Ct^* or 2^–Δ^ ^Δ^ *^Ct^* method (Livak and Schmittgen, 2001). Each cDNA was performed three parallel technical repeats, and the calculated results were visualized as the mean values ± standard error (±SE). The qRT-PCR primers were shown in Supplementary Table 4.

### Statistical Analysis

Statistical differences between WT and *CsPMEI2*/*4*-OE lines under different conditions were analyzed by one-way Analysis of Variance (ANOVA) followed by Tukey’s HSD test and/or Duncan’s test.

## Results

### Identification, Cloning, Bioinformatics Analysis of *CsPMEIs* in Tea Plant

Based on PMEI domain (PF04043), 51 *CsPMEIs* and 2 *CsVIF*/*CIFs* were identified from tea plant genome. The ORF lengths of *CsPMEIs* were varied from 420 to 924 bp, the encoded amino acids were ranged from 139 to 307 aa, the molecular weights (MW) were changed from 15.48 to 34.12 kD, and the theoretical isoelectric points (pIs) were ranged from 3.93 to 9.76 pIs. More than 35 CsPMEIs were predicted to be stable proteins that are located in cytoplasm. 46 CsPMEIs were predicted to contain N-terminal signal peptides and 10 CsPMEIs were predicted to contain transmembrane helices (TMHs) (Table 1). Meanwhile, four *CsPMEIs* genes, named as *CsPMEI1*-*4* (accession number: KU884479, KU884480, KU884481, and KU884482), were cloned based on the ESTs sequences as found in the previous transcriptome data. Alignment analysis results found that CsPMEI1-4 shared more than 99% identities with XP_028079167, XP_028079378, XP_028097250, and XP_028127647, respectively. Amino acid alignment analysis result showed that all of the identified CsPMEIs and CsVIF/CIFs contained four conserved and representative Cys residues except for CsPMEI5 (Supplementary Figure 1).

**TABLE 1:** Basic information of *CsPMEIs*.

Gene name	Accession number	ORF (bp)	AA	MW (kDa)	pI	Instability index	Loc	SignalP	TMHs
CsPMEI1	KU884479	594	197	21.70	4.31	Stable	Nucleus	Yes	No
CsPMEI2	KU884480	591	196	21.52	3.93	Stable	Cytoplasm	Yes	No
CsPMEI3	KU884481	630	209	22.55	9.16	Stable	Nucleus	Yes	1
CsPMEI4	KU884482	639	212	22.86	7.67	Stable	Cytoplasm	Yes	No
CsPMEI5	XP_028119293.1	816	271	28.74	4.5	Unstable	Nucleus	Yes	No
CsPMEI6	XP_028063476.1	504	167	18.27	5.79	Stable	Cytoplasm	Yes	No
CsPMEI7	XP_028078455.1	561	186	20.39	4.37	Stable	Nucleus	Yes	No
CsPMEI8	XP_028080229.1	552	183	19.8	9.1	Stable	Cytoplasm	Yes	No
CsPMEI9	XP_028096826.1	798	265	29.6	5.34	Stable	Cytoplasm	No	1
CsPMEI10	XP_028103971.1	585	194	20.53	6.27	Stable	Nucleus	Yes	No
CsPMEI11	XP_028105374.1	636	211	23	9.32	Unstable	Nucleus	Yes	No
CsPMEI12	XP_028106458.1	615	204	22.25	9.73	Stable	Nucleus	Yes	No
CsPMEI13	XP_028108659.1	639	212	23.28	8.51	Unstable	Nucleus	Yes	No
CsPMEI14	XP_028108660.1	630	209	22.68	9.24	Stable	Cytoplasm	Yes	1
CsPMEI15	XP_028108661.1	639	212	23.14	8.71	Unstable	Nucleus	Yes	No
CsPMEI16	XP_028112715.1	564	187	21.02	9.12	Stable	Nucleus	Yes	1
CsPMEI17	XP_028117331.1	651	216	23.89	4.58	Unstable	Cytoplasm	Yes	No
CsPMEI18	XP_028118374.1	546	181	20.92	4.65	Stable	Nucleus	Yes	No
CsPMEI19	XP_028118383.1	546	181	20.7	4.64	Stable	Cytoplasm	Yes	No
CsPMEI20	XP_028118385.1	546	181	20.96	4.64	Stable	Cytoplasm	Yes	No
CsPMEI21	XP_028118830.1	582	193	21.11	6.42	Stable	Nucleus	Yes	No
CsPMEI22	XP_028118831.1	630	209	22.65	9.36	Stable	Cytoplasm	Yes	No
CsPMEI23	XP_028118832.1	642	213	23.24	8.88	Stable	Cytoplasm	Yes	No
CsPMEI24	XP_028118833.1	618	205	22.21	8.29	Stable	Cytoplasm	Yes	No
CsPMEI25	XP_028118856.1	588	195	20.96	9.25	Stable	Cytoplasm	Yes	No
CsPMEI26	XP_028122572.1	504	167	18.18	5.28	Stable	Cytoplasm	Yes	No
CsPMEI27	XP_028123747.1	630	209	22.68	9.76	Stable	Cytoplasm	Yes	No
CsPMEI28	XP_028123748.1	621	206	21.9	6.09	Stable	Cytoplasm	Yes	No
CsPMEI29	XP_028123765.1	555	184	19.46	5.8	Stable	Chloroplast	No	No
CsPMEI30	XP_028051078.1	630	209	22.7	8.92	Stable	Chloroplast	Yes	No
CsPMEI31	XP_028051784.1	582	193	20.86	9.41	Stable	Cytoplasm	Yes	No
CsPMEI32	XP_028055798.1	504	167	18.17	5.63	Stable	Cytoplasm	Yes	No
CsPMEI33	XP_028056276.1	609	202	22.01	9.47	Unstable	Cytoplasm	Yes	No
CsPMEI34	XP_028061043.1	585	194	21.09	5.59	Stable	Cytoplasm	Yes	No
CsPMEI35	XP_028061148.1	627	208	22.14	8.85	Stable	Cytoplasm	Yes	1
CsPMEI36	XP_028063328.1	420	139	15.48	5.04	Stable	Cytoplasm	No	No
CsPMEI37	XP_028063329.1	558	185	20.33	4.54	Stable	Cytoplasm	Yes	1
CsPMEI38	XP_028063330.1	552	183	20.36	5.27	Stable	Cytoplasm	Yes	1
CsPMEI39	XP_028063858.1	573	190	21.3	6.23	Unstable	Cytoplasm	Yes	1
CsPMEI40	XP_028069427.1	606	201	21.49	4.52	Stable	Cytoplasm	Yes	No
CsPMEI41	XP_028069450.1	621	206	21.96	6.09	Stable	Cytoplasm	Yes	No
CsPMEI42	XP_028073478.1	567	188	20.52	9.14	Stable	Cytoplasm	No	No
CsPMEI43	XP_028073497.1	540	179	19.67	7.66	Stable	Cytoplasm	Yes	No
CsPMEI44	XP_028074709.1	516	171	18.5	6.29	Stable	Cytoplasm	Yes	No
CsPMEI45	XP_028079349.1	504	167	18.27	5.13	Stable	Cytoplasm	Yes	No
CsPMEI46	XP_028079849.1	564	187	21.58	8.82	Unstable	Cytoplasm	Yes	1
CsPMEI47	XP_028081340.1	762	253	27.12	5.11	Stable	Cytoplasm	No	1
CsPMEI48	XP_028082183.1	606	201	21.98	9.33	Stable	Cytoplasm	Yes	No
CsPMEI49	XP_028082302.1	621	206	21.93	6.09	Stable	Cytoplasm	Yes	No
CsPMEI50	XP_028082397.1	540	179	19.6	8.32	Stable	Cytoplasm	Yes	No
CsPMEI51	XP_028090516.1	924	307	34.12	8.92	Unstable	Cytoplasm	Yes	No
CsVIF/CIF1	XP_028053538.1	525	174	18.71	9.23	Stable	Cytoplasm	Yes	No
CsVIF/CIF2	XP_028100701.1	522	173	18.48	9.57	Stable	Cytoplasm	Yes	No

*ORF, opening reading fame; AA, the numbers of amino acid residues; MW, molecule weight; pI, theoretical isoelectric point; Loc, subcellular location; SignalP, signal peptide; TMHs, transmembrane helices.*

**FIGURE 1:**
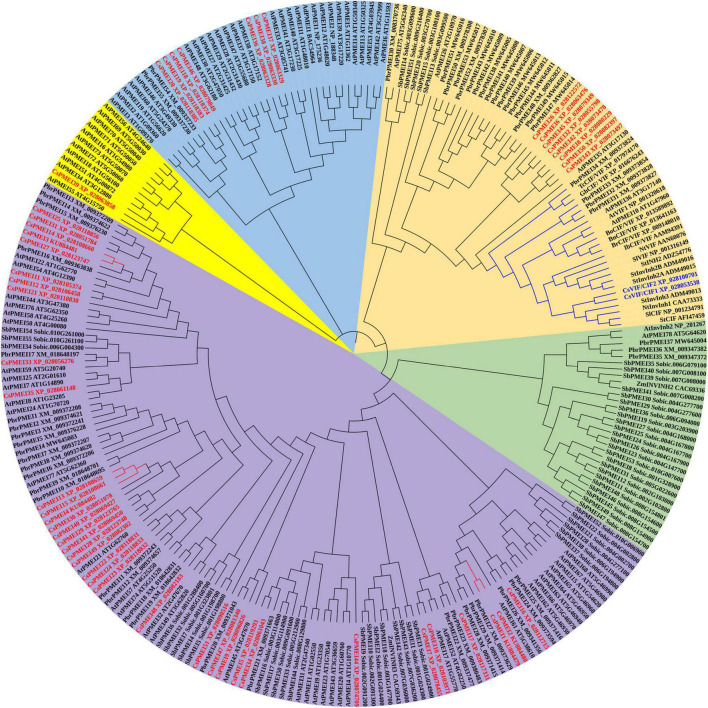
Phylogenetic analysis of *CsPMEIs* and known PMEIs in *Arabidopsis*, *Sorghum bicolor*, pear, and kiwi fruit. A total of 262 PMEI domain contained proteins that were used to construct phylogenetic tree by using MEGA 7.0 software. *CsPMEIs* are highlighted with red color, and different PMEIs subfamilies were covered with different colors.

Phylogenetic analysis result showed that 262 PMEI domain contained proteins were clustered into five clades. However, CsPMEIs and CsVIF/CIFs were only grouped into four clades, among them, 35 of CsPMEIs were clustered into Clade I. In addition, we found that CsVIF/CIFs showed closet relationship with lots of known VIF/CIFs, but most of CsPMEIs showed closet relationship with PbrPMEIs (Figure 1). Moreover, the DNA structure of each *CsPMEI* contains only one exon except for *CsPMEI34* and *CsPMEI42* (Supplementary Figure 2A). *cis*-acting elements prediction results showed that *CsPMEIs* could be regulated by MYB and MYC transcription factors, auxins (GA, MeJ, SA, and ABA etc.) and multiple stresses (drought, cold, and anaerobic etc.) (Supplementary Figure 2B). To verify this conclusion, the expressions of four genes, *CsPMEI1*-*4*, were detected under different conditions. Meanwhile, the functions of *CsPMEI2* and *CsPMEI4* were further explored by means of overexpression technology in *Arabidopsis*.

### Expression Profiles of *CsPMEIs* in Various Tissues, and in Responding to Various Abiotic Stresses in Tea Plant Leaves and Roots

**FIGURE 2:**
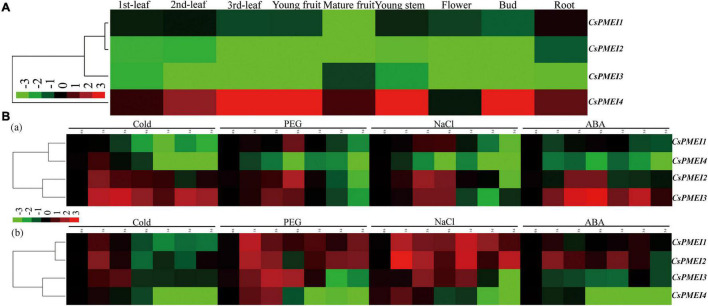
Expression profiles of *CsPMEIs* in tea plant. **(A)** Tissue-specifc expression patterns of *CsPMEIs* in different tea plant tissues. The relative expression levels were calculated by using 2^– Δ^ *^Ct^* method. The colorbar was displayed on the lower-left of the heat map, red and green colors represent higher and lower expression levels, respectively. **(B)** Temporal-spatial expression patterns of *CsPMEIs* both in tea plant mature leaves and roots under various abiotic stresses. **(a)** The expression patterns of *CsPMEIs* in mature leaves; **(b)** The expression patterns of *CsPMEIs* in roots. Samples at 0 h were used as control, and the final results were calculated with 2^– Δ^ ^Δ^ *^Ct^* method. The colorbar was presented on the left side of the heat map, red and green colors represent higher and lower expression levels, respectively. Data are shown as the means ± SE (*n* = 3).

The tissue-specific of *CsPMEI1*-*4* were analyzed in nine different tea plant tissues. As shown in Figure 2A, *CsPMEI1*-*4* showed diverse transcription abundances in various tissues. Among them, *CsPMEI1*/*2* were highly expressed in tender leaves and root, *CsPMEI3* was highly expressed in mature fruit. However, *CsPMEI4* presented the highest expressions in each detected tissue than the other three *CsPMEIs*, especially in young fruit, young stem and bud, suggesting that *CsPMEIs*, especially *CsPMEI4*, widely involved in the vegetative and reproductive processes of tea plant.

Within 5 days of different abiotic stresses treatments, the expressions of *CsPMEIs* showed diverse changes both in tea plant mature leaves and roots at different processing time points (Figure 2B). In mature leaves, *CsPMEI1* transcripts were declined by cold treatment, but induced by drought and NaCl treatments; *CsPMEI2*/*3* transcripts were highly induced by different abiotic stresses within 9 h of treatments, then *CsPMEI2* was reduced by different abiotic stresses until the 5 days of time point; besides, *CsPMEI4* transcripts were significantly decreased within 5 days of different abiotic stresses. In roots, *CsPMEI1* transcripts were highly upregulated by drought and NaCl treatments, but downregulated by cold treatment; *CsPMEI2* transcripts were highly induced by drought, NaCl and ABA stresses within 5 days of different abiotic stresses, except for a few time points; *CsPMEI3* transcripts were increased within 3 h of cold stress and 1 day of drought and NaCl stresses, respectively, but decreased within 5 days of ABA treatment; the mRNA level of *CsPMEI4* was reduced by various abiotic stresses after 3 h of treatments, even though it was induced by drought and NaCl within 3 h of treatments. The above results indicated that *CsPMEI1*-*4* play important roles in dealing with various abiotic stresses in tea plant.

### Differential Expressions of *CsPMEIs* Under Biotic, Cold and Sugars Treatments

**FIGURE 3:**
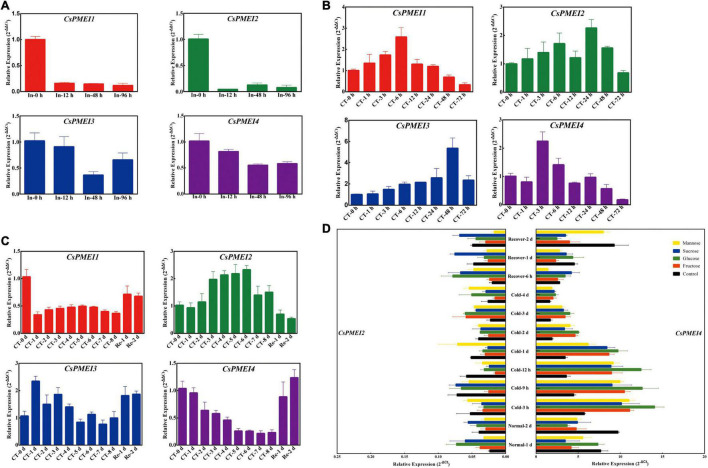
Temporal-spatial expression patterns of *CsPMEIs* in response to biotic, cold and various sguar treatments. **(A)** Expression profiles of *CsPMEIs* under *C. fructicola* infection condition. Samples at 0 h were used as control, and the final results were calculated with 2^– Δ^ ^Δ^ *^Ct^* method. ‘In’ represents infection treatment. Data are shown as the means ± SE (*n* = 3). **(B)** Expression profiles of *CsPMEIs* at one bud and one leaf stage of tea plant under cold condition. Samples at 0 h were used as control, and the final results were calculated with 2^– Δ^ ^Δ^ *^Ct^* method. ‘CT’ represents cold treatment. Data are shown as the means ± SE (*n* = 3). **(C)** Expression profiles of *CsPMEIs* at one bud and three leaves stage of tea plant under cold condition. Samples at 0 h were used as control, and the final results were calculated with 2^– Δ^ ^Δ^ *^Ct^* method. ‘CT’ represents cold treatment. Data are shown as the means ± SE (*n* = 3).**(D)** Expression analysis of *CsPMEIs* in tea plant leaves under different sugar and temperature conditions. All results were calculated by using the 2^– Δ^ *^Ct^* method. Data are shown as the means ± SE (*n* = 3).

Here, we detected the expression patterns of *CsPMEI1*-*4* under *C. fructicola* infection condition. As Figure 3A shown, *CsPMEI1*-*4* transcripts were reduced by *C. fructicola*, especially *CsPMEI1*/*2* were remarkably downregulated by *C*. *fructicola* infection.

Besides, a time-course expressions of *CsPMEI1*-*4* were separately performed in OBOL and OBTL tissues under 4°C condition. As Figures 3B,C shown, the expression patterns of *CsPMEI1*-*4* were varied in OBOL and OBTL. At OBOL stage, *CsPMEI1*/*3* were continuously up-regulated with 6 h and 2 days of cold treatment (CT), respectively; *CsPMEI2* was also induced by CT within 2 days; *CsPMEI4* showed a highest expression at 3 h, and then gradually decreased until 3 days. At OBTL stage, *CsPMEI1*/*4* were gradually downregulated within 8 days of CT, and then recovered to normal expression levels following the temperature increased; in contrast, *CsPMEI2*/*3* were, respectively, upregulated within 8 and 4 days of CT, and also recovered to normal expression levels under normal temperature condition. These results indicated that *CsPMEI1*-*4* had different levels and timings of expressions in different tissues of tea plant under CT condition.

Furthermore, we found exogenous sugars, including Suc, Glc, Fru, and Man independently induced the expression of *CsPMEI2* under normal temperature (NT) condition, while *CsPMEI2* was reduced within 2 days of CT treatment, except for Man that enhanced the expression of *CsPMEI2* under CT treatment. In contrast, the expression of *CsPMEI4* were decreased under NT condition, but remarkably induced by exogenous sugars within 4 days of CT. Specifically, *CsPMEI4* transcripts were increased more than twofold within 1 days of CT (Figure 3D). These results demonstrated that the cold response of *CsPMEI2/4* in tea plant could be mediated by sugar signaling pathway.

### Differential Expressions of *CsPMEIs* During Cold Acclimation and Bud Dormancy Periods

**FIGURE 4:**
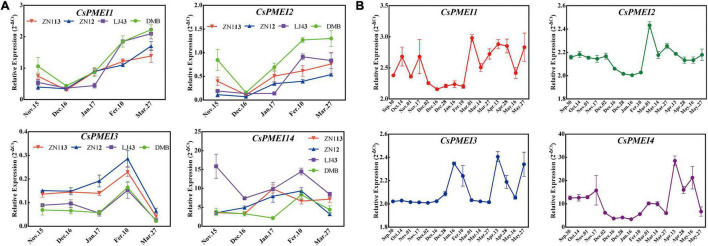
Expression analysis of *CsPMEIs* during CA and bud dormancy periods. **(A)** Expression analysis of *CsPMEIs* in the mature leaves of four-tea cultivars during CA periods. ‘DMB,’ ‘LJ43,’ ‘ZN12,’ and ‘ZN113’ mean ‘DaMianBai,’ ‘LongJing43,’ ‘ZheNong12,’ and ‘ZheNong113’ tea plant cultivar, respectively. The relative expression levels were calculated by using 2^– Δ^ *^Ct^* method. Data are shown as the means ± SE (*n* = 3). **(B)** Expression analysis of *CsPMEIs* in axillary buds of ‘LJ43’ cultivar during bud dormancy period. The relative expression levels were calculated by using 2^– Δ^ *^Ct^* method. Data are shown as the means ± SE (*n* = 3).

Four-tea cultivars with different cold-resistance were used for exploring the expression patterns of *CsPMEI1-4* during CA periods. As Figure 4A shown, *CsPMEIs* showed similar expressions patterns among these four-tea cultivars during CA periods. Briefly, *CsPMEIs* transcripts were decreased during CA periods (from 15 November to 16 December), except for *CsPMEI3*. Subsequently, the expressions of *CsPMEI1*/*2* were increased from 16 December to 27 March. However, *CsPMEI3*/*4* transcripts were firstly increased from 16 December to 10 February, and then decreased with increasing temperature.

We also detected the expressions of *CsPMEI1-4* in axillary buds during bud dormancy period. As Figure 4B shown, *CsPMEI1*/*2*/*4* transcripts were decreased throughout the bud dormancy period, but increased with the dormancy released and at bud sprouting stages. However, the expression of *CsPMEI3* was not changed during para- and endo-dormancy periods (from 30 September to 16 December), but increased during eco-dormancy (after 28 December) and bud sprouting stages (after 27 March). In a word, *CsPMEI1*-*4* involved in the bud dormancy and release of axillary buds in tea plant, but the comprehensive molecular mechanisms need to be further explored.

### Overexpression Analysis of *CsPMEI2* and *CsPMEI4* in Transgenic *Arabidopsis*

#### *CsPMEI4* Decreased the Cold Resistance of Transgenic *Arabidopsis*

**FIGURE 5:**
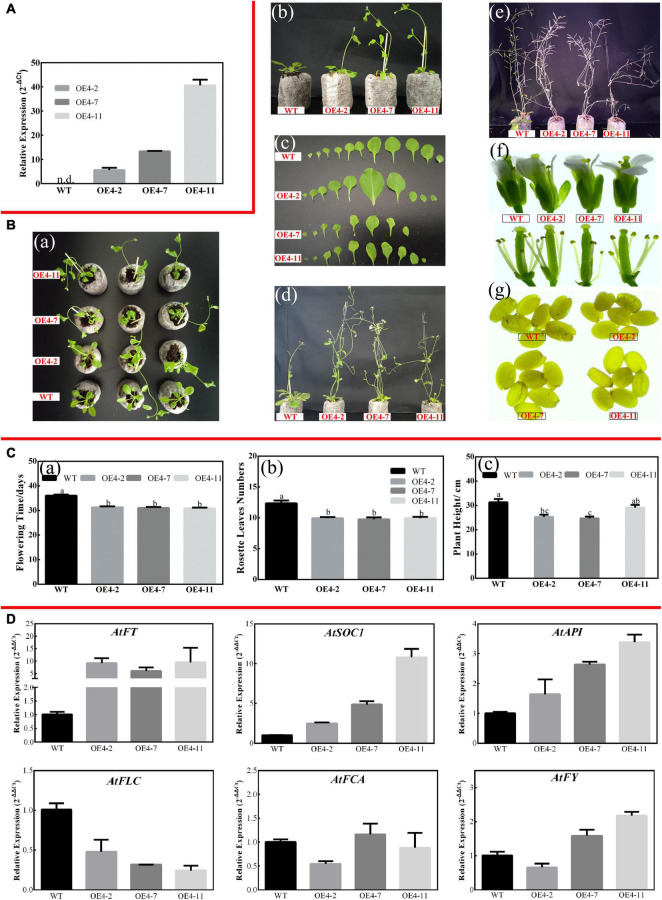
Overexpression of *CsPMEI4* promotes early flowering in transgenic *Arabidopsis* under LD condition. **(A)** The expression of *CsPMEI4* in three *CsPMEI4*-OE lines and WT plants. The relative expression levels were calculated by using 2^– Δ^ *^Ct^* method. Data are shown as the means ± SE (*n* = 3). **(B)** Early flowering phenotypes of *CsPMEI4*-OE lines grown under LD condition. **(a)** Early-flowering phenotype; **(b)** phenotypes of *CsPMEI4*-OE lines and WT plants after 30 d of growth under LD condition; **(c)** leaf size; **(d)** phenotype of *CsPMEI4*-OE lines and WT plants after 40 days of growth under LD condition; **(e)** phenotype of *CsPMEI4*-OE lines and WT plants at senescence stage; **(f)** flowers sizes of WT and *CsPMEI4*-OE lines; **(g)** seed size of WT and *CsPMEI4*-OE lines. **(C)** Flowering time, rosette leaves numbers, and plant height of WT and *CsPMEI4*-OE lines at the just bolting stage. **(a)** Flowering time; **(b)** rosette leaves numbers; **(c)** plant height. **(D)** Expression analysis of flowering-related genes. The final results were calculated with 2^– Δ^ ^Δ^ *^Ct^* method. Data are shown as the means ± SE (*n* = 3).

**FIGURE 6:**
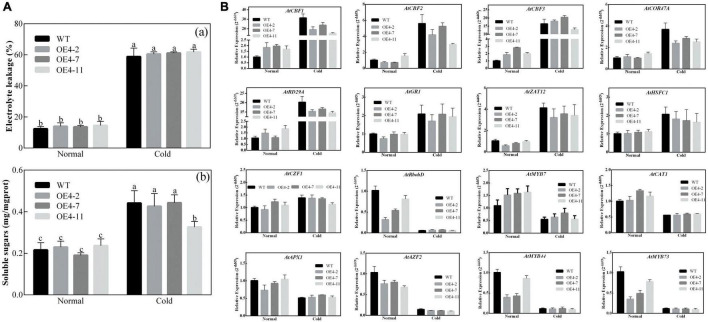
Analysis of EL, TSS contents and cold-related genes transcripts in *CsPMEI4*-OE lines and WT plants. **(A)** EL and TSS contents of *CsPMEI4*-OE lines and WT plants under different temperature conditions. **(a)** Electrolyte leakage, EL; **(b)** total soluble sugar content. Data are shown as the means ± SE (*n* = 3). **(B)** Expression analysis of cold-related genes in *CsPMEI4*-OE lines and WT plants under cold condition. The final results were calculated with 2^– Δ^ ^Δ^ *^Ct^* method. Data are shown as the means ± SE (*n* = 3).

As described in Figures 2, 3, *CsPMEI4* was significantly repressed by CT in tea plant. Here, we further explored the cold resistance of *CsPMEI4*-OE lines under CT conditions. Three independent *CsPMEI4*-OE lines (OE4-2, OE4-7, and OE4-11) with different transcription abundances were separately selected to perform the following experiments (Figure 5A). Unfortunately, the phenotypes had not shown obvious difference between *CsPMEI4*-OE lines and WT plants under CT condition (data not shown). However, *CsPMEI4*-OE lines showed higher EL, but lower TSS contents than the WT plants even though no significant difference detected between them (Figure 6A). Besides, the expression levels of many cold-induced genes (*AtCBF1*-*3*, *AtCOR47A*, *AtRD29A*, *AtGR1*, *AtZAT12*, *AtHSFC1*, and *AtCZF1*) in *CsPMEI4*-OE lines were lower than that in WT plants under CT condition. In addition, the transcript abundances of four cold-repressors (*AtAZF* and *AtMYB7*/*44*/*73*), two antioxidant-related genes (*AtCAT1* and *AtAPX1*), and one ROS-producing enzyme NADPH oxidase gene (*AtRbohD*) were decreased both in *CsPMEI4*-OE lines and WT plants under CT condition (Figure 6B). These results suggested that overexpression of *CsPMEI4* slightly reduced the cold-resistance of transgenic *Arabidopsis*.

#### *CsPMEI2* and *CsPMEI4* Promote Early Flowering in Transgenic *Arabidopsis* Both Under Long-Day and Short-Day Conditions

Apart from influencing cold tolerance of transgenic *Arabidopsis*, an obvious difference was observed on the flowering time between *CsPMEI2*/*4*-OE lines and WT plants.

**FIGURE 7:**
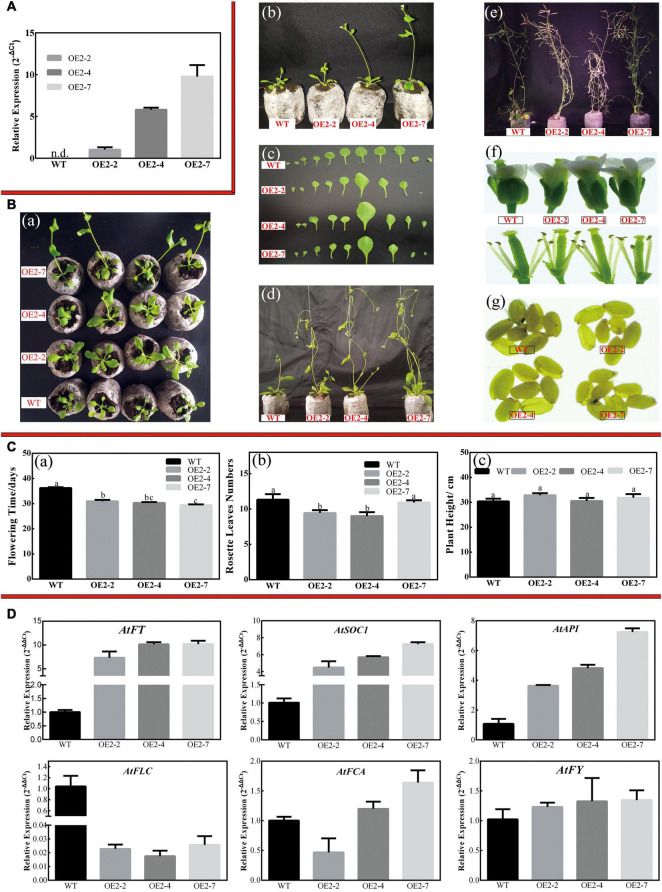
Overexpression of *CsPMEI2* promotes early flowering in transgenic *Arabidopsis* under LD condition. **(A)** The expression of *CsPMEI2* in three *CsPMEI2*-OE lines and WT plants. The relative expression levels were calculated by using 2^– Δ^ *^Ct^* method. Data are shown as the means ± SE (*n* = 3). **(B)** Early flowering phenotypes of *CsPMEI2*-OE lines grown under LD condition. **(a)** Early-flowering phenotype; **(b)** phenotypes of *CsPMEI2*-OE lines and WT plants after 29 days of growth under LD condition; **(c)** leaf size; **(d)** phenotypes of *CsPMEI2*-OE lines and WT plants after 38 days of growth under LD condition; **(e)** phenotypes of *CsPMEI2*-OE lines and WT plants at senescence stage; **(f)** flowers of WT and *CsPMEI2*-OE lines; **(g)** seed size of WT and *CsPMEI2*-OE lines. **(C)** Flowering time, rosette leaves, and plant height of WT and *CsPMEI2*-OE lines at the just bolting stage. **(a)** Flowering time; **(b)** rosette leaves numbers; **(c)** plant height. **(D)** Expression analysis of flowering-related genes. The final results were calculated with 2^– Δ^ ^Δ^ *^Ct^* method. Data are shown as the means ± SE (*n* = 3).

Three independent *CsPMEI2*-OE lines (OE2-2, OE2-4, and OE2-7) and *CsPMEI4*-OE lines (OE4-2, OE4-7, and OE4-11) with different transcription abundances were separately selected to perform the following experiments (Figures 5A, 7A). Under LD condition, both *CsPMEI2*-OE lines and *CsPMEI4*-OE lines present early flowering than the WT plants (Figures 5B, 7B). Concretely, the WT plants require *c*. 36 days from seeds sterilization to floral bud formation, while *CsPMEI2*-OE lines and *CsPMEI4*-OE lines require *c*. 30 and 31 days, respectively (Figures 5C, 7C). It is now clear that early-flowering plants usually possess less leaves during flowering periods (Xiong et al., 2019). The similar phenotypes were also detected in *CsPMEI2*/*4*-OE lines, where we found that both *CsPMEI2*-OE lines and *CsPMEI4*-OE lines formed *c*. 9 rosette leaves, while the WT plants formed *c*. 12 rosette leaves as the first flower opened (Figures 5C, 7C). In addition, both *CsPMEI2*-OE lines and *CsPMEI4*-OE lines showed earlier senescence phenotypes than the WT plants (Figures 5B, 7B). As compared to WT plants, the plant heights of *CsPMEI2*-OE lines were taller, while *CsPMEI4*-OE lines were shorter (Figures 5C, 7C). Besides, the flower sizes of *CsPMEI2*-OE lines are longer and slender, and the pistils are smaller than the WT plants, while the flower structures (numbers of sepals, petals, and stamens) and the sizes of seeds have not shown significant distances among them (Figure 7B). However, the flower sizes, the flower structures (numbers of sepals, petals, stamens, and pistils) and the sizes of seeds of *CsPMEI4*-OE lines are similar to the WT plants (Figure 5B).

**FIGURE 8:**
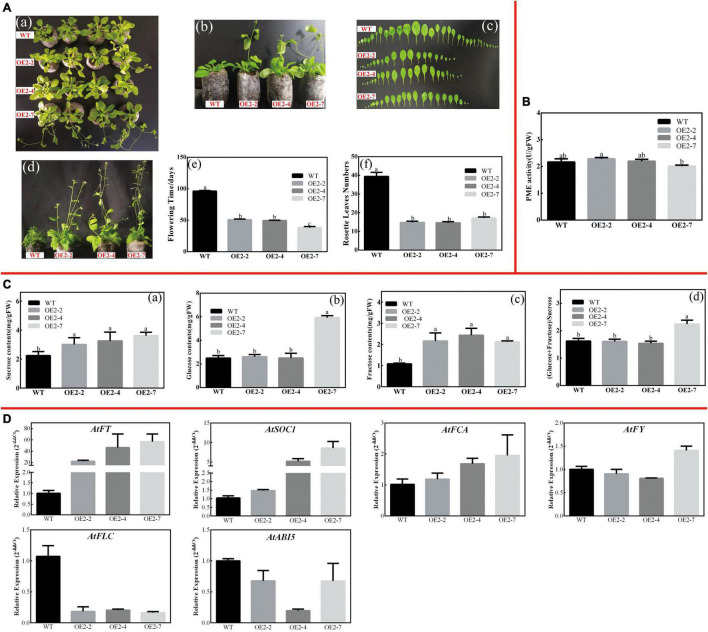
Overexpression of *CsPMEI2* promotes early flowering in transgenic *Arabidopsis* under SD condition. **(A)** Early flowering phenotypes of *CsPMEI2*-OE lines grown under SD condition. **(a)** Early-flowering phenotype; **(b)** phenotypes of *CsPMEI2*-OE lines and WT plants after 38 d of growth under SD condition; **(c)** leaf size; **(d)** phenotypes of *CsPMEI2*-OE lines and WT plants after 96 days of growth under SD condition; **(e)** flowering time; **(f)** rosette leaves numbers. **(B)** PME activities of WT and *CsPMEI2*-OE lines under SD condition. **(C)** Sugar contents of WT and *CsPMEI2*-OE lines under SD condition. **(a)** Suc contents; **(b)** Glu contents; **(c)** Fru contents; **(d)** the ratio of hexose/Suc. **(D)** Expression analysis of flowering-related genes. The final results were calculated with 2^– Δ^ ^Δ^ *^Ct^* method. Data are shown as the means ± SE (*n* = 3).

**FIGURE 9:**
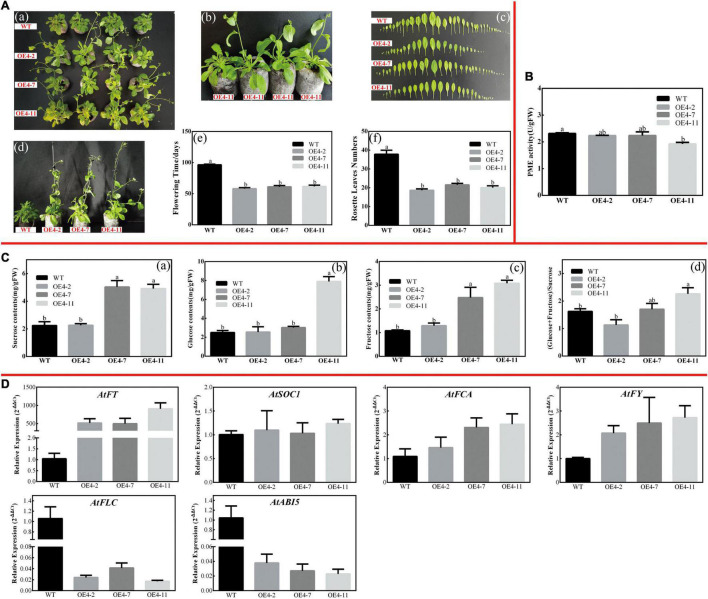
Overexpression of *CsPMEI4* promotes early flowering in transgenic *Arabidopsis* under SD condition. **(A)** Early flowering phenotypes of *CsPMEI4*-OE lines grown under SD condition. **(a)** Early-flowering phenotype; **(b)** phenotypes of *CsPMEI4*-OE lines and WT plants after 58 d of growth under SD condition; **(c)** leaf size; **(d)** phenotypes of *CsPMEI4*-OE lines and WT plants after 96 days of growth under SD condition; **(e)** flowering time; **(f)** rosette leaves numbers. **(B)** PME activities of WT and *CsPMEI4*-OE lines under SD condition. **(C)** Sugar contents of WT and *CsPMEI4*-OE lines under SD condition. **(a)** Suc contents; **(b)** Glu contents; **(c)** Fru contents; **(d)** the ratio of hexose/Suc. **(D)** Expression analysis of flowering-related genes. The final results were calculated with 2^– Δ^ ^Δ^ *^Ct^* method. Data are shown as the means ± SE (*n* = 3).

Similarly, *CsPMEI2*/*4*-OE lines also promote early flowering in transgenic *Arabidopsis* under SD condition as compared to WT plants. Concretely, *CsPMEI2*-OE lines and *CsPMEI4*-OE lines, respectively, need *c.* 38 days and *c.* 58 days from seeds sterilization to the floral bud formation, while WT plants need more than 3 months to bloom. Correspondingly, *CsPMEI2*-OE lines and *CsPMEI4*-OE lines formed *c. 15* and *c. 20* rosette leaves, while the WT plants formed *c*. 38 rosette leaves as the first flower bloomed (Figures 8A, 9A). These results indicate that *CsPMEI2* and *CsPMEI4* mediating flowering control may be independent of photoperiod pathway.

#### *CsPMEI2* and *CsPMEI4* Affect PME Activities and Sugar Contents in Transgenic *Arabidopsis* Under Short-Day Condition

Under SD condition, the seedlings were sampled to validate the effects of *CsPMEI2* and *CsPMEI4* on PME activities of transgenic *Arabidopsis*. As Figures 8B, 9B show, the PME activities seem to be not significantly inhibited by the exogenous overexpression of *CsPMEI2* or *CsPMEI4*, except for the highest expressions OE-lines (OE2-7 and OE4-11). In addition, the sugars contents, including Suc, Glu, and Fru were increased both in *CsPMEI2*-OE and *CsPMEI4*-OE lines. Particularly, Suc and Fru contents in OE lines were significantly higher than that in WT plants, and the ratios of (Glu + Fru)/Suc were also higher both in *CsPMEI2*-OE and *CsPMEI4*-OE lines than the WT plants, except for OE4-2 (Figures 8C, 9C). These results indicate that *CsPMEI2* and *CsPMEI4* mediated flowering was associated with the changes of sugars contents.

#### *CsPMEI2* and *CsPMEI4* Affect Multiple Flowering-Relate Genes Transcripts in Transgenic *Arabidopsis*

To explore the molecular mechanisms of early flowering phenotype of *CsPMEI2*-OE lines and *CsPMEI4*-OE lines, we further conducted the expression levels of many flowering responsive genes.

Under LD condition, the expressions of two floral integrators, *FLOWERING LOCUS T* (*FT*) and *SUPPRESSOR OF OVEREXPRESSION OF CONSTANS 1* (*SOC1*), were remarkably increased, while a key flowering repressor gene, *FLOWERING LOCUS C* (*FLC*), was significantly inhibited both in *CsPMEI2*-OE lines and *CsPMEI4*-OE lines as compared to WT plants. In addition, three floral meristem identity genes, *APETALA1* (*AP1*), *FLOWERING CONTROL LOCAL A* (*FCA*), and *FLOWERING LOCUS T* (*FY*) were also upregulated both in *CsPMEI2*-OE lines and *CsPMEI4*-OE lines (Figures 5D, 7D). Similarly, under SD condition, the expressions of *FT*, *SOC1*, *FCA*, and *FY* were upregulated both in *CsPMEI2*-OE lines and *CsPMEI4*-OE lines, especially for *FT* gene, which is induced at least *c.* 20-fold and *c.* 400-fold in *CsPMEI2*-OE lines and *CsPMEI4*-OE lines, respectively. Meanwhile, the mRNA levels of *FLC* and an ABA stimulated positive factors, *ABSCISIC ACID-INSENSITIVE 5* (*ABI5*) were decreased both in *CsPMEI2*-OE lines and *CsPMEI4*-OE lines (Figures 8D, 9D). These results suggested that *CsPMEI2* and *CsPMEI4* promoting early flowering may partially depend on ABA-dependent pathway. This inference was supported by ABA treatments, where we found the flowering times of *CsPMEI2*-OE and *CsPMEI4*-OE lines were delayed by the application of exogenous ABA, even though the development of all plants retarded by ABA treatment (Supplementary Figure 3).

.

## Discussion

### *CsPMEIs* Exist in Large Multigene Family Exhibiting Diverse Expression Patterns in Tea Plant

At post-translation level, the activity of PME is antagonized by PMEI. As a type of small molecular protein, PMEI is encoded by large multigene family in various plant species, such as *Arabidopsis* (Müller et al., 2013), *B. campestris* (Liu et al., 2018b), rice (Nguyen et al., 2016), pear (Zhu et al., 2021), etc. It is now clear that both PMEI and INH share moderate sequence homology, in particular the presence of four conserved Cys residues that form two disulfide bridges (S–S) (Camardella et al., 2000; Hothorn et al., 2004). However, PMEI and INH are selectively targeted toward PMEs and plant acid INVs (CWIN and VIN), respectively. For INH, a conserved amino acid motif ‘PKF’ (Proline, Lysine, and Phenylalanine) has been demonstrated as core motif that directly targets the active site of the CWIN/VIN (Hothorn et al., 2010). Herein, 53 genes with intact and conserved PMEI domains were identified from ‘ShuChaZao’ tea plant cultivar genome. Bioinformatics analysis results showed that most of *CsPMEIs* contain one exon on DNA level encoding a type of 21 KD proteins with an N-signal peptide. Phylogenetic analysis result showed that CsPMEIs were clustered into four clades, which are similar to the phylogenetic results of PbrPMEIs (Zhu et al., 2021) and BcPMEIs (Liu et al., 2018b). In addition, we found 2 CsVIF/CIFs showed closet relationships with many known INHs, and only these two CsVIF/CIFs contain the conserved ‘PKF’ motif that directly targets the INV active site (Supplementary Figure 1). These two genes have been cloned and sequenced recently, and their functions will be further explored in future. Besides, phylogenetic analysis result showed that PMEIs and INHs were clearly clustered into different subgroups, most of known INHs, including CsVIF/CIFs, were clustered into subgroup I, while CsPMEI2/4 were clustered into subgroup III (Supplementary Figure 4A). This result was further verified by the amino acid alignment result, where we found all members of subgroup I contain the conserved ‘PKF’ motif, but PMEIs do not contain this motif (Supplementary Figure 4B). Collectively, we considered that 51 *CsPMEIs* and 2 *CsVIF*/*CIFs* genes were identified from ‘ShuChaZao’ tea plant cultivar genome, and CsPMEI2/4 belonged to PMEI family members.

In recent years, lots of experiments have validated that PMEIs function in various biological processes, including seed germination (Müller et al., 2013), pollen growth (Zhu et al., 2021), fertility (Andres-Robin et al., 2020), organ formation and separation, fruit ripening (Srivastava et al., 2012), biotic and abiotic stress response (An et al., 2008; Volpi et al., 2011; Lionetti et al., 2017). Recently, many *PMEIs* genes involved in abiotic stresses also have been identified and explored in plants, while their molecular regulation mechanisms are rarely studied. Microarray data analysis results showed that many *AtPMEIs* were differentially expressed in response to different abiotic stresses (Liu et al., 2018b). Similarly, the meta-transcriptional analysis results found that lots of *OsPMEIs* were transcriptionally up- or down-regulated by abiotic and biotic stresses (Nguyen et al., 2016). Exogenous overexpression of *CaPMEI* enhanced drought tolerance and alleviated the sensitive to mannitol-induced osmotic stress in transgenic *Arabidopsis* seedlings as compared to WT plants (An et al., 2008). Herein, *CsPMEI1*-*4* exist transcriptional diversity and functional division. In various tea plant tissues, *CsPMEI4* transcripts were remarkably accumulated in each detected tissue, suggesting that it may play an important role in response to various biological processes in tea plant. Besides, *CsPMEI1-4* were up- or down-regulated by various abiotic stresses in a certain period of treatment time, suggesting that CsPMEIs possess functional specialization, and a balance mechanism of pectin regulation exists in tea plant in response to abiotic stresses. This assumption was also supported by the results of Table 1, which indicated that the PME activity of tea plant was co-regulated by CsPMEI superfamily genes at the post-translational level, rather than by several CsPMEI genes.

Previous studies found that pectin contents, PME activity and the low-methylated pectin contents increased in the leaves of winter oil-seed rape during CA period, while a contrary tendency exhibited as the de-acclimation proceeded (Solecka et al., 2008). Similarly, Baldwin et al. (2014) found that the levels of arabinose, galactose, galacturonic acid and xylose residues changed in *Pisum sativum* under CA condition. Moreover, the degree of DM increased after 10–20 days of CA and 2 days of frost treatment as compared to non-CA plants. Meanwhile, the PME activity increased after 10–20 days of CA, but decreased after 2 days of frost treatment in frost-tolerant genotype ‘Champagne’ as compared to non-CA ‘Champagne,’ which indicated that methylesterification of pectins contributes to improving frost-tolerance of pea during CA period (Baldwin et al., 2014). In this study, we found that the expressions of *CsPMEI1*-*2* declined in tea plant leaves during CA period, while increased with the process of de-acclimation. Meanwhile, *CsPMEI1*-*4* transcripts decreased throughout the bud dormancy period, but increased with the breaking dormancy and the bud sprouting. These results inferred that the PME activity, pectin contents and the methyl-esterification degree of pectins may be dynamically changed by temperature both in tea plant leaves and axillary buds. However, this assumption still needs to be further verified due to PME activity was inhibited by a large superfamily members of CsPMEIs as showed in Table 1. Therefore, the expression analysis of all CsPMEIs, the assay of PME activity and pectins contents should be further performed in order to extensively explore the specific regulation mechanism of CsPMEIs involved in freezing tolerance of tea plant.

In addition to responding to abiotic stress, *PMEIs* transcripts are also affected by hormones. As Srivastava et al. (2012) demonstrated that the expression of a ripening related gene, *MaPMEI*, relies on ethylene-dependent pathway indirectly during banana ripening. Similarly, the transcription levels of a wheat *PMEI* gene, *TaPMEI*, were regulated by SA, ABA, and MeJA in leaves, stem and root (Hong et al., 2010). Meanwhile, the expression levels of *CaPMEI1* were not only induced by abiotic stresses, but also by ABA, SA, ethylene and MeJA at various time intervals, suggesting that *CaPMEI1* may be mediated the early active defense responses to bacterial pathogen infection and exogenous hormones treatment (An et al., 2008). Herein, we found the transcription abundance of *CsPMEI2*/*3* were strongly induced after 9 h of ABA treatment, while *CsPMEI1*/*4* were significantly declined during ABA treatment period, suggesting that *CsPMEI2*/*3* may be involved in hormone-related signaling pathways associated with defense-responsive.

At present, lots of evidences have verified that PMEIs are involved in plant defense against pathogen infection. CaPMEI1 exhibited basal disease resistance against a variety of plant pathogenic fungi, overexpression of *CaPMEI1* enhanced the anti-fungal ability in transgenic *Arabidopsis*, which indicated that the increased PMEI activity may cause the decrease accessibility of fungal pectin degrading-enzymes and thus enhance disease resistance of transgenic *Arabidopsis* (An et al., 2008). Similar results were also found in *AtPMEI*-*1* or *AtPMEI*-*2* overexpressed *Arabidopsis* lines after infected by *B*. *cinerea* (Lionetti et al., 2007). However, it has been reported that increasing PME activity also improved the ability of pathogen immunity. During pattern-triggered immunity and after inoculation with necrotrophic fungus and bacterial hemibiotroph in *Arabidopsis*, the PME activity increased, but the degree of pectin methylesterification decreased. Further research found that pathogen-induced PME activity was dependent on JA signaling pathway. Mutating the selected *pme* gene resulted in high sensitivity to pathogen, but the total PME activity was not influenced in *pme* mutants, suggesting that PME enhancing the immunity responsive was not determined by total PME activity, but by some specific effect of PMEs, such as methyl-esterification degree of pectins (Bethke et al., 2014). For tea plant, lots of differentially expressed metabolites (e.g., gluconic acid, fatty acid, amino acid, organic acid, etc.) have been identified after being inoculated with *C*. *camelliae*. Meanwhile, the contents of JA and IAA were significantly increased, accompanied by the transcription accumulation of a pathogenesis-related protein 4 (*PR4*) in JA signaling pathway, which indicated that tea plant-*Colletotrichum* interaction may be mainly mediated by JA signaling pathway (Lu et al., 2020). Herein, all four *CsPMEIs* transcripts were significantly inhibited by *C*. *fructicola* infection in tea plant, suggesting that the low transcription abundance of *CsPMEIs* may result in the decrease of PMEI activity, and thus increasing PME activity may change the methyl-esterification degree of pectins depending on JA signaling pathway.

### Exogenous Overexpression of *CsPMEIs* Affects Cold-Resistance and Flowering Time in Transgenic *Arabidopsis*

It has been reported that the PME activity, pectins content and the methyl-esterification degree of pectins were dynamically changed under cold conditions (Solecka et al., 2008; Baldwin et al., 2014). In present study, there are no obvious phenotypic differences observed between *CsPMEI4*-OE lines and WT plants under cold condition. However, the higher EL, but the lower TSS contents and the lower expressions of many cold-induced genes were found in *CsPMEI4*-OE lines as compared with WT plants, suggesting that *CsPMEI4* may play negative roles in responding to cold stress in transgenic *Arabidopsis*. A similar result was also obtained by Chen et al. (2018), where they found overexpression of *CbPMEI* from *Chorispora bungeana* or *PMEI13* (AT5G62360) from *Arabidopsis* decreased PME activity and the content of low-DM pectins in transgenic *Arabidopsis*. Furthermore, both *CbPMEI*-OE and *PMEI13*-OE lines are sensitive to freezing stress, while tolerant to salt stress. Expression analysis results showed that *CbPMEI* and *PMEI13* mediated freezing tolerance may be independent on *CBF* pathway due to *CBFs* and *CORs* transcripts were not consistently declined in transgenic lines (Chen et al., 2018). However, the molecular and genetic mechanisms of PMEIs involved in abiotic stress tolerance still remain poorly understood, how the increased PMEI activity affects abiotic resistance needs to be further explored.

It is now clear that PMEIs are involved in pollen tube growth through inhibiting PME activity and hence affect cell wall stability (Rockel et al., 2008). A *PMEI* gene, *BoPMEI*, has been demonstrated as a pollen-specific gene, which is critical to pollen tube growth. Antisense expression of *BoPMEIl* in *Arabidopsis* suppressed the transcriptions of an orthologous gene, *Atlgl0770*, which resulted in partial male sterility and decreased seed set (Zhang et al., 2010). In *B. campestris*, many *BcPMEIs* transcripts were highly expressed in inflorescences, in particular 10 *BcPMEIs* transcripts were specifically expressed during flower development periods (Liu et al., 2018b). Moreover, mature transcripts of two *PMEI* genes, *Tdpmei2.1* and *Tdpmei2.2*, were predominantly detected in floral organs of durum wheat, which indicated that *Tdpmei2.1* and *Tdpmei2.2* play key roles in flower development, in particular in anther and pollen development (Rocchi et al., 2012). Since many studies have demonstrated that PMEIs are involved in the reproductive process of plant, the roles in promoting flowering have not been reported. In the present study, both *CsPMEI2*-OE and *CsPMEI4*-OE lines exhibited early flowering phenotypes as compared to WT plants both under LD and SD conditions. Meanwhile, we found the PME activities were slightly decreased under SD condition. It is well known that the inhibition of PME activity leads to an increase in methylesterification of cell wall HGs, but the changes to cell walls were spatially regulated (Müller et al., 2013). Here, we failed to detect the methylesterification contents as the technology restriction. Instead, we found the degradation productions contents of pectins, including Suc, Glu and Fru were significantly increased both in *CsPMEI2*-OE and *CsPMEI4*-OE lines, suggesting that overexpression of *CsPMEI2*/*4* induced the changes of carbohydrate metabolism, and hence affected reproductive process. However, the specific regulation mechanisms need to be further explored. Actually, a similar result was also found by Müller et al. (2013), where they demonstrated that overexpression of *AtPMEI5* in *Arabidopsis* resulted in lower PME activity and higher contents of neutral sugars (i.e., Glu, fucose, and rhamnose) in cell wall, but the levels of uronic acids were not significantly changed as compared with WT plants.

In terms of flowering, at least four major pathways, including autonomous, gibberellin, photoperiod, and vernalization pathways, have been identified to participate in floral promotion (Wang et al., 2012; Jing et al., 2020). During flowering period, two flowering pathway integrators (*FT* and *SOC1*), which are commonly regulated by these four pathways, will activate two floral meristem identity genes (*AP1* and *LFY*) to promote the formation of floral meristems (Wang et al., 2012). However, such integrators are negatively regulated by a central upstream regulator, *FLC*, which encodes a MADS box transcription factor and mediates the autonomous or vernalization pathways (Michaels and Amasino, 2001). The transcription accumulation of genes involved in the autonomous pathway (e.g., *FCA* and *FY*) would suppress the expression of *FLC* through chromatin or RNA modification (Wang et al., 2012). As expected, the transcriptions of *FT*, *SOC1*, and *AP1* and some genes involved in the autonomous pathway, including *FCA* and *FY*, were accumulated, while the expression of *FLC* was declined both in *CsPMEI2*-OE and *CsPMEI4*-OE lines as compared to WT plants under LD and SD condition. A similar phenotype was also found in *CsUGT85A53*-OE transgenic *Arabidopsis*. The expressions of *FT*, *SOC1*, *AP1*, *LFY*, *FY*, and *FCA* were increased, while the mRNA level of *FLC* was repressed in *CsUGT85A53*-OE lines. Further research found that overexpression of *CsUGT85A53* increased the DNA methylation levels of *FLC*, but decreased the free ABA contents via ABA glucosylation in transgenic *Arabidopsis*. However, the early flowering phenotype was restored by the application of exogenous ABA (Jing et al., 2020). In this study, we found the expression of an activator of *FLC* gene, *ABI5*, was also declined both in *CsPMEI2*-OE and *CsPMEI4*-OE lines. However, exogenous ABA treatment did not completely inhibit early flowering phenomenon of *CsPMEI2*-OE and *CsPMEI4*-OE lines, suggesting that the early flowering phenomenon in transgenic *Arabidopsis* may be partially dependent on ABA signaling pathway, and the changes of carbohydrate metabolism may also contribute to this phenomenon. Still, the detailed molecular mechanism of the roles of *CsPMEI2*/*4* in flowering needs to be deeply explored.

## Data Availability Statement

The datasets presented in this study can be found in online repositories. The names of the repository/repositories and accession number(s) can be found in the article/Supplementary Material.

## Author Contributions

WQ conceived and designed the experiments. WQ and HW wrote the original draft. HW and BL performed the experiments. YW, NL, LW, and XH sampled the materials. SH, BL, and WQ analyzed the results and performed figures. ZD and YY reviewed and edited the manuscript. All authors read and approved the final manuscript.

## Conflict of Interest

The authors declare that the research was conducted in the absence of any commercial or financial relationships that could be construed as a potential conflict of interest.

## Publisher’s Note

All claims expressed in this article are solely those of the authors and do not necessarily represent those of their affiliated organizations, or those of the publisher, the editors and the reviewers. Any product that may be evaluated in this article, or claim that may be made by its manufacturer, is not guaranteed or endorsed by the publisher.

## Footnotes

1
http://pfam.xfam.org/

2
http://smart.embl-heidelberg.de/

3
https://www.ncbi.nlm.nih.gov/cdd/advanced

4
https://itol.embl.de/

5
http://gsds.cbi.pku.edu.cn/

6
http://bioinformatics.psb.ugent.be/webtools/plantcare/html/

## Supplementary Material

Supplementary Figure 1 Conserved domains analysis of CsPMEIs and CsVIF/CIFs. Four conserved and representative Cys (C) residues were contained in the red boxes, respectively.

Supplementary Figure 2 The exon–intron structures, and *cis*-acting elements of *CsPMEIs*. **(A)** Exon–intron structure of *CsPMEIs*. The coding sequence and the corresponding genomic sequence of each *CsPMEI* were compared by using GSDS2.0. Blue boxes represent untranslated upstream/downstream regions, green double sided wedges represent exons, and black lines indicate introns. **(B)** The *cis*-acting regulatory elements of *CsPMEIs*. 2,000-bp upstream non-coding region sequences of each *CsPMEI* gene, except for *CsPMEI3*/*20*, were used to predict *cis*-acting elements, and different colored blocks represent different elements.

Supplementary Figure 3 Phenotypes, flowering time and rosette leaves numbers of *CsPMEI2/4*-OE lines under ABA treatment condition. **(A)** Phenotypes, flowering time and rosette leaves numbers of *CsPMEI2*-OE lines under ABA treatment condition. **(a)** Phenotypes of *CsPMEI2*-OE lines and WT plants after 31 days of growth under ABA treatment condition; **(b)** phenotypes of *CsPMEI2*-OE lines and WT plants after 41 days of growth under ABA treatment condition; **(c)** phenotypes of *CsPMEI2*-OE lines and WT plants after 51 days of growth under ABA treatment condition; **(d)** flowering time; **(e)** rosette leaves numbers. **(B)** Phenotypes, flowering time and rosette leaves numbers of *CsPMEI4*-OE lines under ABA treatment condition. **(a)** Phenotypes of *CsPMEI4*-OE lines and WT plants after 35 days of growth under ABA treatment condition; **(b)** phenotypes of *CsPMEI4*-OE lines and WT plants after 41 days of growth under ABA treatment condition; **(c)** phenotypes of *CsPMEI4*-OE lines and WT plants after 51 days of growth under ABA treatment condition; **(d)** flowering time; **(e)** rosette leaves numbers.

Supplementary Figure 4 Phylogenetic and conserved domains analysis of INHs and PMEIs. **(A)** A total of 30 INHs and PMEIs were used to construct phylogenetic tree by using MEGA 7.0 software. CsVIF/CIFs highlighted with red squares and CsPMEIs are highlighted with red triangle, and different subgroups were covered with different colors. **(B)** Conserved domains analysis of INHs and PMEIs. The conserved ‘PKF’ motif was contained in the blue box, and the four conserved and representative Cys (C) residues were contained in the red boxes, respectively.

Supplementary Table 1 Nutrient solution formulation.

Supplementary Table 2 All sequences used to construct phylogenetic tree.

Supplementary Table 3 Primer sequences used in ORF of *CsPMEI2*/*4* cloning and vector construction.

Supplementary Table 4 Primers information used in qRT-PCR detection.
