# Supplementary figures and images for: Genome-Wide Identification of the PMEI Gene Family in Tea Plant and Functional Analysis of CsPMEI2 and CsPMEI4 Through Ectopic Overexpression
Source: Front Plant Sci. 2022 Jan 27;12:807514. doi: 10.3389/fpls.2021.807514 (PMC8829431; doi:10.3389/fpls.2021.807514)

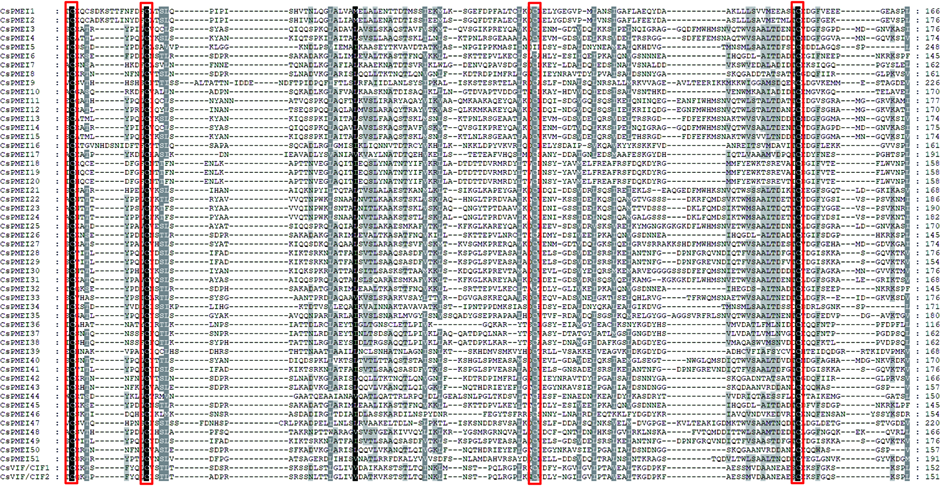

Supplement: Supplementary Figure 1 — Conserved domains analysis of CsPMEIs and CsVIF/CIFs. Four conserved and representative Cys (C) residues were contained in the red boxes, respectively. [file Image_1.TIF]

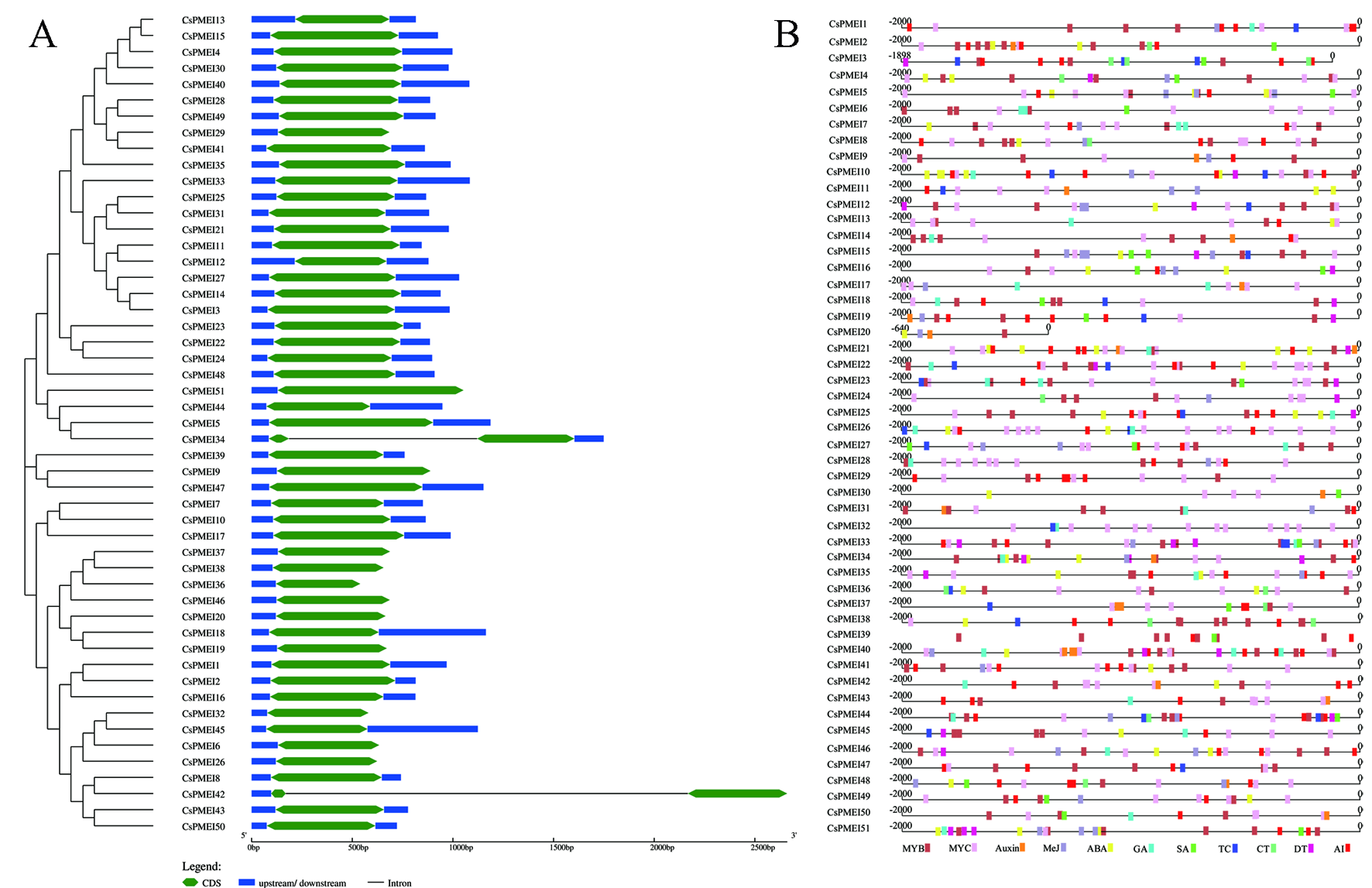

Supplement: Supplementary Figure 2 — The exon–intron structures, and cis-acting elements of CsPMEIs. (A) Exon–intron structure of CsPMEIs. The coding sequence and the corresponding genomic sequence of each CsPMEI were compared by using GSDS2.0. Blue boxes represent untranslated upstream/downstream regions, green double sided wedges represent exons, and black lines indicate introns. (B) The cis-acting regulatory elements of CsPMEIs. 2,000-bp upstream non-coding region sequences of each CsPMEI gene, except for CsPMEI3/20, were used to predict cis-acting elements, and different colored blocks represent different elements. [file Image_2.TIF]

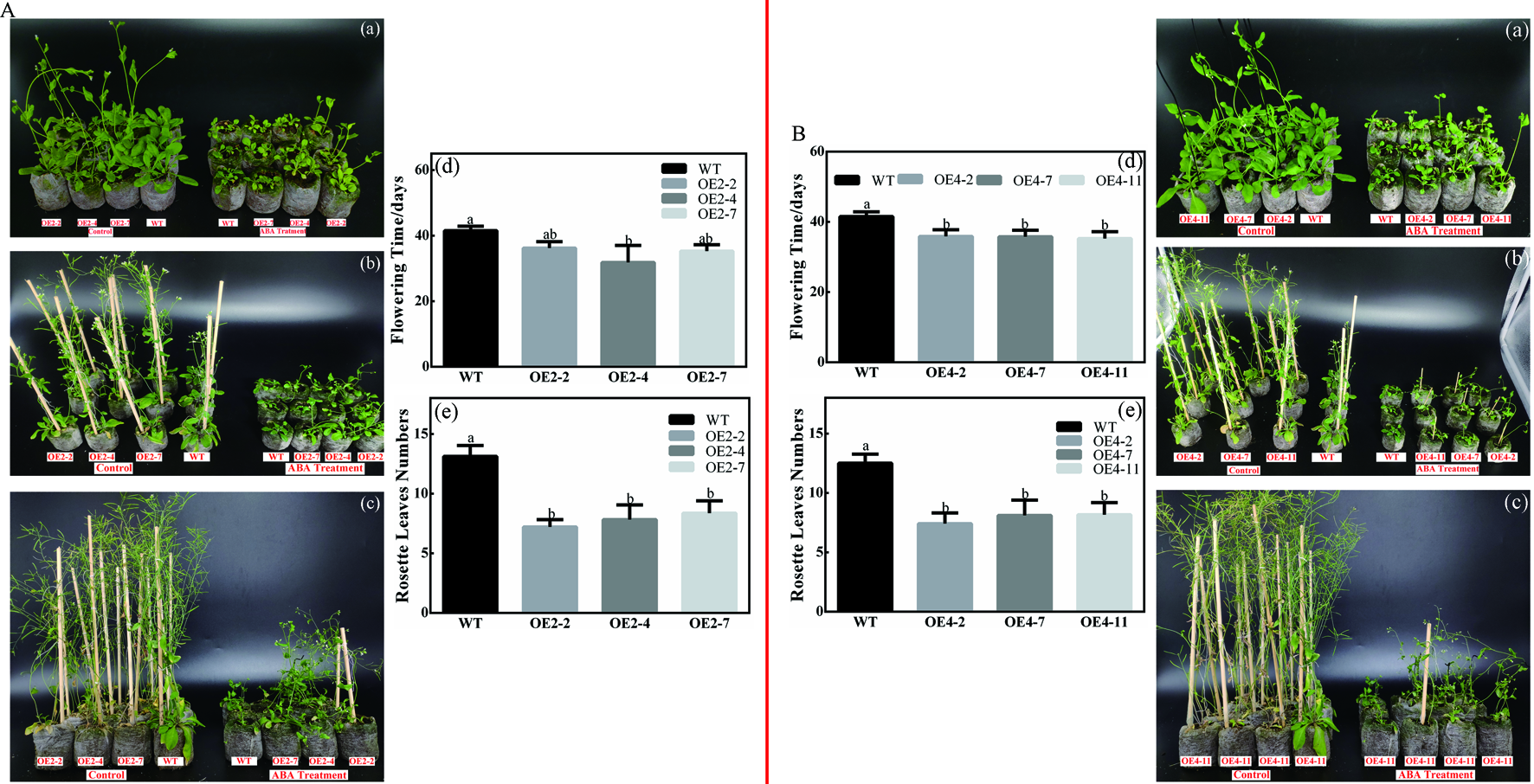

Supplement: Supplementary Figure 3 — Phenotypes, flowering time and rosette leaves numbers of CsPMEI2/4-OE lines under ABA treatment condition. (A) Phenotypes, flowering time and rosette leaves numbers of CsPMEI2-OE lines under ABA treatment condition. (a) Phenotypes of CsPMEI2-OE lines and WT plants after 31 days of growth under ABA treatment condition; (b) phenotypes of CsPMEI2-OE lines and WT plants after 41 days of growth under ABA treatment condition; (c) phenotypes of CsPMEI2-OE lines and WT plants after 51 days of growth under ABA treatment condition; (d) flowering time; (e) rosette leaves numbers. (B) Phenotypes, flowering time and rosette leaves numbers of CsPMEI4-OE lines under ABA treatment condition. (a) Phenotypes of CsPMEI4-OE lines and WT plants after 35 days of growth under ABA treatment condition; (b) phenotypes of CsPMEI4-OE lines and WT plants after 41 days of growth under ABA treatment condition; (c) phenotypes of CsPMEI4-OE lines and WT plants after 51 days of growth under ABA treatment condition; (d) flowering time; (e) rosette leaves numbers. [file Image_3.TIF]

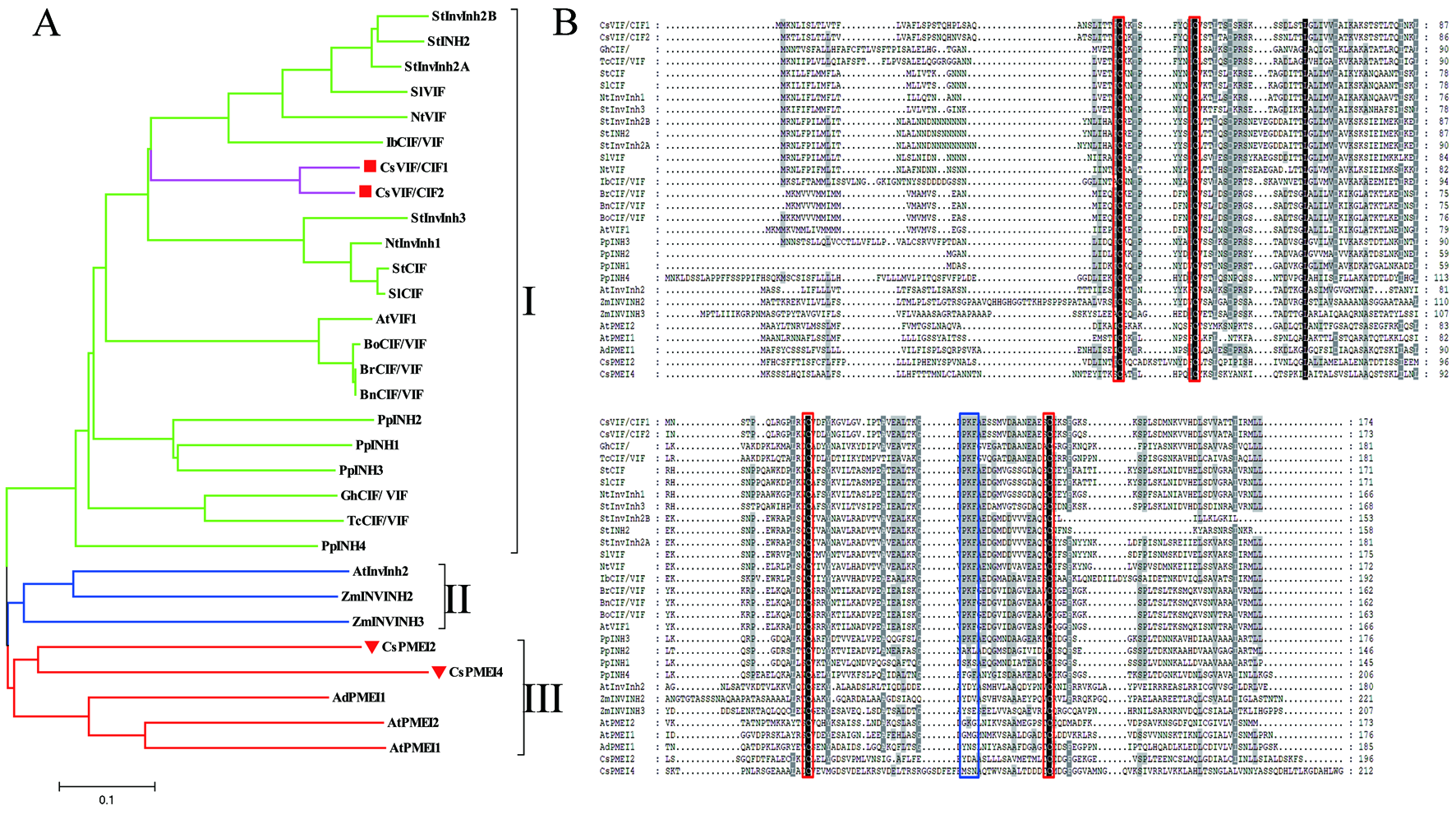

Supplement: Supplementary Figure 4 — Phylogenetic and conserved domains analysis of INHs and PMEIs. (A) A total of 30 INHs and PMEIs were used to construct phylogenetic tree by using MEGA 7.0 software. CsVIF/CIFs highlighted with red squares and CsPMEIs are highlighted with red triangle, and different subgroups were covered with different colors. (B) Conserved domains analysis of INHs and PMEIs. The conserved ‘PKF’ motif was contained in the blue box, and the four conserved and representative Cys (C) residues were contained in the red boxes, respectively. [file Image_4.TIF]
