# Supplementary material for: Genome-Wide Identification of the PMEI Gene Family in Tea Plant and Functional Analysis of CsPMEI2 and CsPMEI4 Through Ectopic Overexpression
Source: Front Plant Sci. 2022 Jan 27;12:807514. doi: 10.3389/fpls.2021.807514 (PMC8829431; doi:10.3389/fpls.2021.807514)
Supplement: Supplementary Table 1 — Nutrient solution formulation. [file Data_Sheet_1.docx]

**Supplementary Table 1** Nutrient solution formulation.

| **Elements** | **Compounds** | **Concentration** |
| --- | --- | --- |
| **Macroelements**  **(mmolL^-1^)** | CaCl_2_ | 0.53 |
|  | NH_4_NO_3_ | 2 |
|  | KH_2_PO_4_ | 0.07 |
|  | K_2_SO_4_ | 0.6 |
|  | MgSO_4_·7H_2_O | 0.67 |
|  | A1_2_(SO_4_)_3_ ·l8H_2_O | 0.07 |
| **Microelements**  **(μmolL^-1^)** | EDTA·Na_2_Fe | 4.2 |
|  | CuSO_4_.5 H_2_O | 0.13 |
|  | ZnSO_4_·7 H_2_O | 0.67 |
|  | H_3_BO_3_ | 7 |
|  | MnSO_4_·H_2_O | 1 |
|  | (NH_4_)_6_Mo_7_O_24_.4 H_2_O | 0.33 |

**Supplementary Table 3** Primer sequences used in ORF of CsPMEI2/4 cloning and vector construction.

| Gene name | Primer sequence |
| --- | --- |
| CsPMEI1F | 5′-ATGTGGCACTGCTCTTTCTTCACCA-3′ |
| CsPMEI1R | 5′-TTAGCGGTTCAATAGGACTTTAGA-3′ |
| CsPMEI2F | 5′-ATGAAAGACCAACGAGTTTTC-3′ |
| CsPMEI2R | 5′-TCAAGAGAACTTGGAATCCAAGGC-3′ |
| CsPMEI3F | 5′-ATGGCAAGACTTGGAACTTTCTTGC-3′ |
| CsPMEI3R | 5′-TTAAGGCATGTTCTTAGTTCCAGA-3′ |
| CsPMEI4F | 5′-ATGAAAAGTTCATCATTACACCA-3′ |
| CsPMEI4R | 5′-TCAGCCCCAGAGATGGGCATCACC-3′ |
| CsPMEI2OE-F | 5′-CACCATGAAAGACCAACGAGTTTTC-3′ |
| CsPMEI2OE-R | 5′-AGAGAACTTGGAATCCAAGGC-3′ |
| CsPMEI4OE-F | 5′-CACCATGAAAAGTTCATCATTACACC-3′ |
| CsPMEI4OE-R | 5′-GCCCCAGAGATGGGCATCACCTTTCA-3′ |

**Supplementary Table 4** Primer information used in qRT-PCR detection.

| **ID** | **Gene name** | **Forward/Reverse** | **Primer sequence (5' to 3')** |
| --- | --- | --- | --- |
| AT4G25490 | *AtCBF1* | Forward | GGAGACAATGTTTGGGATGC |
|  |  | Reverse | TTAGTAACTCCAAAGCGACACG |
| AT4G25470 | *AtCBF2* | Forward | GACGTGTCCTTATGGAGCTATTAAAA |
|  |  | Reverse | TTACCATTTACATTCGTTTCTCACAAC |
| AT4G25480 | *AtCBF3* | Forward | TTCCGTCCGTACAGTGGAAT |
|  |  | Reverse | AACTCCATAACGATACGTCGTC |
| AT5G59820 | *AtZAT12* | Forward | GTGCGAGTCACAAGAAGCCTAACA |
|  |  | Reverse | GCGACGACGTTTTCACCTTCTTCA |
| AT1G20440 | *AtCOR47* | Forward | CAGTGTCGGAGAGTGTGGTG |
|  |  | Reverse | ACAGCTGGTGAATCCTCTGC |
| At2G40140 | *AtCZF1* | Forward | GCCTTGTCCCGAGTTTCGTA |
|  |  | Reverse | TGCGCGTACTCACACGAATC |
| AT1G07890 | *AtAPX1* | Forward | TCGCATGGCACTCTGCTGGAAC |
|  |  | Reverse | CACCAGTAACTTCAACGGCCAC |
| AT5G47910 | *AtRbohD* | Forward | GCCGGCCTAAACGTGCGTCCAA |
|  |  | Reverse | GCGGGAGGCGTTCTTGATGCGT |
| AT1G20630 | *AtCAT1* | Forward | ACCTGTTGGTCGCTTGGTCTTGA |
|  |  | Reverse | GGTGAGCACATTTAGGGGCATTA |
| AT5G52310 | *AtRD29A* | Forward | GCCGAGAAACTTCAGATTGG |
|  |  | Reverse | CCATTCCTCCTCCTCCTTTC |
| AT3G24170 | *AtGR1* | Forward | TGTAGGAGATGCCACAAACCG |
|  |  | Reverse | CACTACAGCTAGTGGTGGTATG |
| AT3G24520 | *AtHSFC1* | Forward | CCGGATGGGTGGATTGTTCCTATGAC |
|  |  | Reverse | GTTGAATTCGAGAGCATCGACTTCGC |
| AT3G19580 | *AtAZF2* | Forward | TACGAAGGCAACCTCGGCGG |
|  |  | Reverse | CGTGCTCGACACGCTTCCAC |
| AT2G16720 | *At**MYB7* | Forward | TCGCTGCGGTAAAAGCTGCC |
|  |  | Reverse | AGACCACTTGTTGCCTAGGAGGC |
| AT5G67300 | *AtMYB44* | Forward | AATGGGGAAGTCTTTTCCCGGTAACG |
|  |  | Reverse | CATTGTTCCGTTGCATCTCCGTCATG |
| AT4G37260 | *AtMYB73* | Forward | TGAGGAGTTACATGGCGGATT |
|  |  | Reverse | CGCCGCCAGAACTACTACCA |
| AT5G10140 | *AtFLC* | Forward | CTAGCCAGATGGAGAATAATCATCATG |
|  |  | Reverse | TTAAGGTGGCTAATTAAGTAGTGGGAG |
| AT1G65480 | *AtFT* | Forward | ATGTCTATAAATATAAGAGAGC |
|  |  | Reverse | CTAAAGTCTTCTTCCTCCGCAG |
| AT2G45660 | *AtSOC1* | Forward | AGCTGCAGAAAACGAGAAGCTCTCTG |
|  |  | Reverse | GGGCTACTCTCTTCATCACCTCTTCC |
| AT4G16280 | *AtFCA* | Forward | CCCGTTAGGTGGTTATGGTGTTCC |
|  |  | Reverse | TTGGTTTGGTTGCTGCATAGACTG |
| AT1G69120 | *AtAP1* | Forward | CATGGGTGGTCTGTATCAAGAAGAT |
|  |  | Reverse | CATGCGGCGAAGCAGCCAAGGTT |
| AT2G36270 | *AtABI5* | Forward | TCGACAAGGCTCTTTGACAC |
|  |  | Reverse | ATTACCGCTACCACCACCTC |
| At5G19510 | *AtEF* | Forward | GCTGTTCGTGGTGTTGAGATGC |
|  |  | Reverse | AGGCTCTGAGGTGAGGAAGTCT |
| KU884479 | *CsPMEI1* | Forward | ACTGCACCGAACCGGCTCTCCCAT |
|  |  | Reverse | CGAGTGCAAGCCCTTGAAGGTTTGTG |
| KU884480 | *CsPMEI2* | Forward | CTGCTCCTGATACCGCATGAAAATTAC |
|  |  | Reverse | TGAGTGCAAGTCCTTGGAGGTTCACA |
| KU884481 | *CsPMEI3* | Forward | CGAAACAACTCGCACAAGC |
|  |  | Reverse | TGCCACACGAAGTCCTGA |
| KU884482 | *CsPMEI4* | Forward | GACTCGTAGTAGAGGCGGCT |
|  |  | Reverse | CCCACCAAACCCATCCAT |
| GAAC01052498.1 | *CsPTB* | Forward | TGACCAAGCACACTCCACACTATCG |
|  |  | Reverse | TGCCCCCTTATCATCATCCACAA |
